# Evidence for genetic correlation between appendix and inflammatory bowel disease: A bidirectional Mendelian randomization study

**DOI:** 10.1371/journal.pone.0342541

**Published:** 2026-02-11

**Authors:** Dan Liu, Wanyue Dan, Bin Yan, Lihua Peng, Fei Pan

**Affiliations:** 1 School of Medicine, Nankai University, Tianjin, China; 2 Department of Gastroenterology and Hepatology, the First Medical Center, Chinese PLA General Hospital, Beijing, China; Tehran University of Medical Sciences, IRAN, ISLAMIC REPUBLIC OF

## Abstract

**Background:**

Observational studies highlighted a link between the appendix and inflammatory bowel disease (IBD), but proving causality has been difficult due to the lack of a clear temporal sequence.

**Methods:**

This research used a two-sample bidirectional univariable Mendelian randomization (MR), multivariable MR and linkage disequilibrium score regression (LDSC) analyses to explore the relationship between acute appendicitis, appendectomy, and IBD. Eligible instrumental variables were screened from previous genome-wide association studies (GWAS) of European ancestry for analysis. The inverse variance-weighted method was used for the primary analysis. Sensitivity analyses were used to detect and correct pleiotropy. LDSC analysis determined SNP-based heritability (h2) for acute appendicitis, IBD, Crohn’s disease (CD), and ulcerative colitis (UC). Following that, cross-trait LDSC analysis assessed genetic correlations (rg) between these traits using GWAS summary data.

**Results:**

Genetically predicted UC was significantly associated with a lower risk of acute appendicitis (OR = 0.933, 95% CI 0.911–0.957, p < 0.001) and appendectomy (OR = 0.954, 95% CI 0.932–0.976, p < 0.001). Conversely, no causal effect was observed from acute appendicitis or appendectomy on IBD, UC, or CD. While a suggestive association was noted for CD with appendectomy in univariable analysis, it did not remain significant after multivariable adjustment for the influence of UC. A significant negative genetic correlation further supported the inverse relationship between UC and acute appendicitis (rg = −0.205, p = 0.005).

**Conclusions:**

In conclusion, genetically predicted UC was causally associated with a decreased risk of acute appendicitis and appendectomy, but neither acute appendicitis nor appendectomy has a causal impact on the risk of IBD, UC, or CD. These findings suggest that ulcerative colitis (UC) may confer a protective effect against the development of acute appendicitis, offering valuable insights into the shared genetic architecture and potential biological mechanisms underlying these conditions.

## Introduction

Inflammatory bowel disease (IBD), a chronic relapsing inflammatory disorder of the gastrointestinal tract, has witnessed a global surge in incidence over recent decades [1]. It primarily consists of two main subtypes, Crohn’s disease (CD) and ulcerative colitis (UC), with distinct pathophysiological patterns. CD manifests as transmural inflammation across any gastrointestinal segment, whereas UC is typically limited to the mucosal surface of the colon in a continuous pattern [2]. Though genetic predisposition, environmental factors, and gut dysbiosis are implicated in IBD pathogenesis [3], the pathogenesis remains poorly understood, especially the appendix’s role.

The involvement of the appendix in the pathogenesis of UC has been postulated, but its nature and underlying mechanisms of this association remain elusive [4,5]. Observational studies have demonstrated a notable decrease in the incidence of UC among individuals diagnosed with appendicitis [6–9]. Furthermore, several investigations have highlighted the potential of appendectomy to reduce the risk of UC and enhance clinical outcomes [10–15]. Despite extensive research on the correlation between appendicitis, appendectomy, and UC, questions remain over whether they are causally related. This is in part due to heterogeneity among existing studies and the inherent limitations of observational studies. While most studies suggest a beneficial effect of appendicitis or appendectomy on UC, the results of emerging meta-analyses are inconsistent [16–19]. Moreover, whether this association is attributed to appendectomy or appendicitis, remains conflicting [20]. Further mechanistic evidence that the appendix is involved in the pathogenesis of UC is ambiguous [5]. Additionally, there is a lack of research utilizing causal inference approaches to address the inherent biases present in these studies, such as confounding and reverse causation. These discrepancies predominantly originate from three inherent limitations of observational studies: unmeasured confounding, reverse causation, and phenotypic heterogeneity. Conventional epidemiological methods remain inadequate to resolve these issues, thereby necessitating Mendelian randomization analysis for rigorous causal inference.

Mendelian randomization (MR) overcomes these constraints by leveraging genetic variants as instrumental variables to simulate randomized controlled trials [21]. This study employed bidirectional univariable and multivariable MR, combined with linkage disequilibrium score regression (LDSC), to investigate potential causal relationships between acute appendicitis, appendectomy, and IBD subtypes, including CD and UC, using GWAS summary statistics from individuals of European ancestry.

## Methods

For the purpose of this research, de-identified participant study data that were made available to the public and ethical standards committee-approved were used. In this research, no additional ethical approval was needed.

### Study design

**Fig 1:**
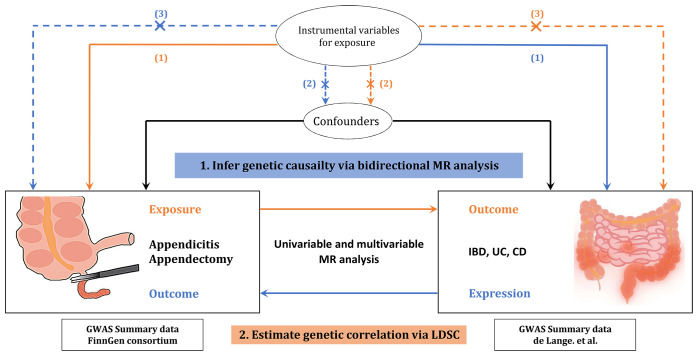
Outline of study design and statistical analysis performed in this study. MR and LDSC analyses were performed using European population based GWAS summary statistics. In MR analyses, the association among IVs, exposure, and outcome are presented as orange lines, whereas blue lines indicate these associations in the reverse direction. While dashed lines indicate links that can break MR assumptions (not present in this MR study), solid lines represent the associations that have been established. IVs, instrumental variables; MR, Mendelian randomization; IBD, inflammatory bowel disease; UC, ulcerative colitis; CD, Crohn’s disease; LDSC, linkage disequilibrium score regression.

This is a two-sample bidirectional, UVMR and MVMR study. Single nucleotide polymorphisms (SNPs) significantly associated with exposures were selected as instrumental variables (IVs). Three important MR hypotheses were adopted: (1) the genetic IVs are strongly associated with exposures, including IBD and its subtypes, acute appendicitis, and appendectomy; (2) the IVs are not associated with confounding factors; and (3) the IVs only affect the outcome via exposure [22]. A schematic diagram of the study design and adopted MR assumption is depicted in Fig 1. The need for informed consent or ethical approval was waived because all statistics in this study were based on publicly available datasets.

### Data sources

Eligible GWAS meta-analysis datasets for exposures and outcomes were searched and retrieved from IEU openGWAS and the FinnGen Biobank Analysis Consortium R9 release. The genetic background of the study samples was predominantly of European ancestry to minimize ethnicity-related confounding factors. The summary statistics for IBD (n = 25,042 cases and 34,915 controls), CD (n = 12,194 cases and 28,072 controls), and UC (n = 12,366 cases and 33,609 controls) were obtained from a large GWAS meta-analysis conducted by de Lange et al. in 2017 [23]. IBD and its subtypes were diagnosed using recognized endoscopic, histopathological, and radiological criteria [23]. The summary statistics for acute appendicitis and appendectomy were extracted from the FinnGen Biobank Analysis Consortium R9 release, which comprised 28,745 patients with acute appendicitis and 346,283 controls, as well as 26,013 individuals who underwent appendectomy and 346,283 controls.

### Selection of instrumental variables

The SNPs that were significantly associated with IBD and its subtypes with genome-wide significance (p < 5 × 10^−8^) were screened as potential IVs. To limit linkage disequilibrium and ensure the independence of the genetic variations used as IVs, a clumping algorithm with SNPs as a standard parameter (r^2^ < 0.001 and window size = 10,000 kb) was used [24,25]. To minimize horizontal pleiotropy, the remaining SNPs were screened using PhenoScanner [26]. SNPs showing strong associations (p < 5 × 10 ⁻ ⁶) with known confounders or with outcome-related traits (appendicitis or appendectomy) were excluded. This filtering step was applied to prevent violation of the exclusion-restriction assumption, whereby genetic instruments should influence the outcome only through the exposure of interest. Importantly, SNPs were not excluded on the basis of inferred causal effects on the outcome itself, but only when evidence suggested potential pleiotropic pathways independent of IBD. Using PhenoScanner, rs6584282, rs7911680, and rs10748782 were identified as having strong associations with monocyte count, a marker of systemic immune cell composition that may influence appendicitis risk independently of IBD, and were therefore excluded as pleiotropic instruments. The power of each instrument was assessed using the F-statistic, and SNPs with an F-statistic of <10 were regarded as “weak instruments” and excluded from MR analysis [27]. The F-statistic was calculated by the following equation: $F=\frac{(N-\text{2}){\times R}^{\text{2}}}{\left(\text{1}-R^{\text{2}}\right)}$. R^2^ denotes the variance of exposure explained by each IV, and N represents the sample size [28].

A similar procedure was used to select IVs for acute appendicitis and appendectomy.

### Statistical analysis

Analyses were done using the statistical software R (version 4.2.2). For most of the part, the packages “TwoSampleMR”, “MVMR”, “data.table”, “dplyr”, “ggplot2 and “ldscr” were used.

Forward UVMR analyses were conducted to evaluate the effect of acute appendicitis and appendectomy on IBD, CD, and UC. Subsequently, reverse UVMR was performed using genetic variants associated with IBD, CD, and UC to estimate their causal effects on acute appendicitis and appendectomy. IVs with direct effects on the outcome were excluded if their p value was less than 1 × 10^−6^. Additionally, to ensure data integrity, palindromic SNPs were removed by harmonizing the exposure and outcome datasets.

Regarding the overlap of genetic instruments between UC and CD, MVMR was employed to assess the independent effect of each trait. Then, to assess the heterogeneity of SNPs, we conducted Cochran’s Q test. In cases where significant heterogeneity was detected (p < 0.05), the IVW random-effects model was employed. Conversely, the IVW fixed-effects model was utilized when no significant heterogeneity was observed.

We used multiple complementary MR approaches to quantify the effect of exposure on outcome susceptibility, including the IVW, weighted median (WM), MR-Egger regression, and MR-pleiotropy residual sum and outlier analysis (MR-PRESSO) methods. Each method has a different assumption regarding the reliability of IVs. Among them, IVW was regarded as the primary analysis method. Because all the variables were binary, odds ratios (ORs) with 95% confidence intervals (CIs) were used to interpret the causal estimates. Bonferroni correction was adopted to prevent false-positive results, and a two-sided P-value of 0.0083 (0.05/6) was considered statistically significant. Associations with P-values of 0.0083–0.05 were suggestive significance. Scatterplots and forest plots were constructed to visualize the MR results.

### Pleiotropy and sensitivity analyses

The heterogeneity of the selected IVs was quantified using the Cochrane’s Q test. The random-effects IVW was performed to generate more conservative and reliable estimates. Furthermore, funnel plots were constructed to determine the presence of heterogeneous IVs. MR-Egger regression analysis was performed to check horizontal pleiotropy. The intercept term of the MR-Egger regression indicated the pleiotropic effect of IVs [29]. The robustness and consistency of the results were determined using the leave-one-out method. Additionally, the MR-PRESSO method was conducted to remove outliers and correct for horizontal pleiotropy [30].

### Heritability and genetic correlation analyses

LDSC analysis was performed to calculate SNP-based heritability (h2) for acute appendicitis, IBD, CD, and UC respectively. Cross-trait LDSC analysis was then conducted to evaluate the genetic correlations (rg) between acute appendicitis and IBD and its subtypes using GWAS summary data.

## Results

### Effects of acute appendicitis and appendectomy on IBD and its main subtypes

**Fig 2:**
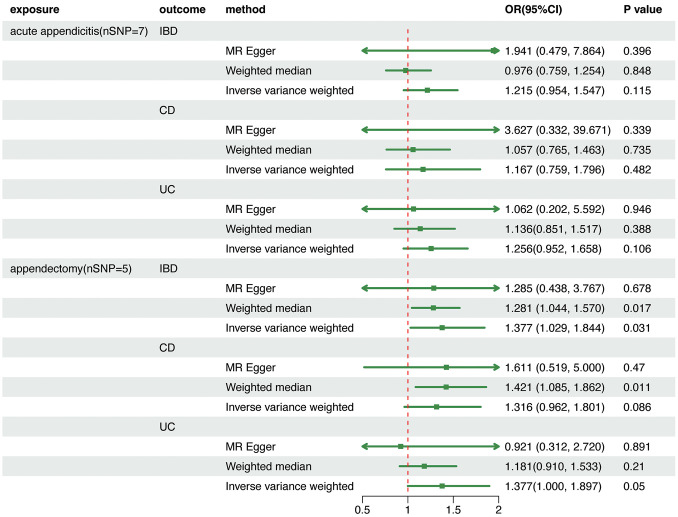
Associations among genetically predisposed acute appendicitis, appendectomy, and the risk of IBD, CD, and UC in UVMR analyses. Estimated odds ratios (ORs) represent the effect of per log-OR increase in appendicitis and appendectomy on IBD, CD, and UC using the random-effect IVW method. MR, Mendelian randomization; IBD, inflammatory bowel disease; UC, ulcerative colitis; CD, Crohn’s disease.

Following the abovementioned filtering procedures, 12 and 7 significant (p < 5 × 10^−8^) and independent (r^2^ < 0.001 and window size = 10,000 kb) IVs for acute appendicitis and appendectomy, were selected respectively. The F-statistics for all IVs were >10, indicating strong IVs. S1-S3 Tables provide detailed information about genetic IVs. Analyses revealed significant heterogeneity (all p < 0.05). Therefore, a random-effect model was used in IVW analysis; the results showed no significant association between acute appendicitis and IBD (all p > 0.05), as shown in Fig 2 and S4 Table. A suggestive association between appendectomy and IBD (OR = 1.377, 95% CI = 1.029–1.537, p = 0.031) was found. Further analyses found a suggestive association between appendectomy and UC (OR = 1.377, 95% CI = 1.000–1.897, p = 0.05). This may indicate that appendectomy could be linked to an increased risk of IBD, a possibility that appears primarily attributable to UC (S5 Table and S1 Fig). Sensitivity analyses were illustrated by the forest plots and leave-one-out plots (S2-S4 Figs).

### Causal effects of IBD and its subtypes on acute appendicitis and appendectomy

Using the abovementioned screening procedures, 117, 62, and 89 significant (p < 5 × 10^−8^) and independent (r^2^ < 0.001 and window size = 10,000 kb) SNPs were chosen as IVs for IBD, UC, and CD, respectively. F-statistics for all IVs were >10, indicating no weak IV bias (S1 Table). Individual IVs for IBD, CD, and UC are detailed in S6-S8 Tables.

**Fig 3:**
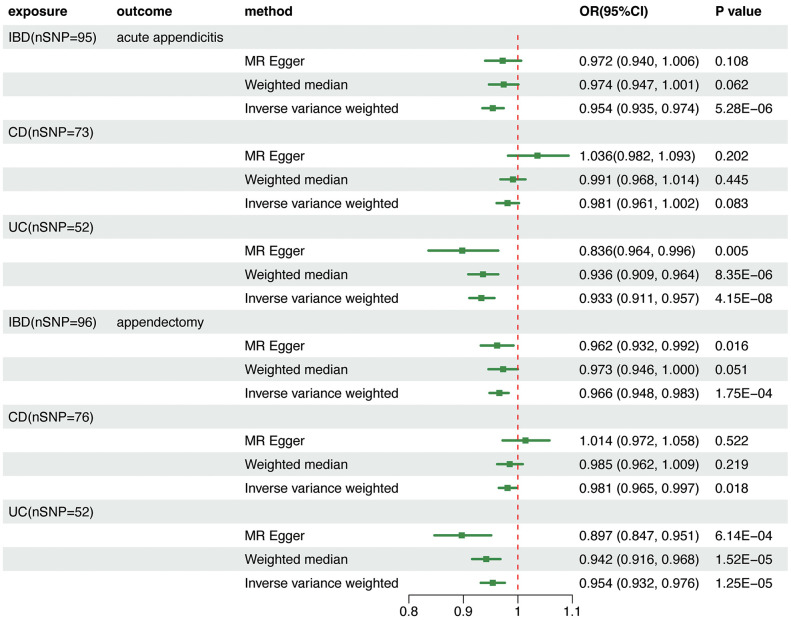
Association between genetically predisposed IBD, CD, and UC and the risk of acute appendicitis and appendectomy in UVMR analyses. Estimated odds ratios (ORs) represent the effect of per log-OR increase in IBD, CD, and UC on appendicitis and appendectomy using the random-effect IVW method. MR, Mendelian randomization; IBD, inflammatory bowel disease; UC, ulcerative colitis; CD, Crohn’s disease.

**Fig 4:**
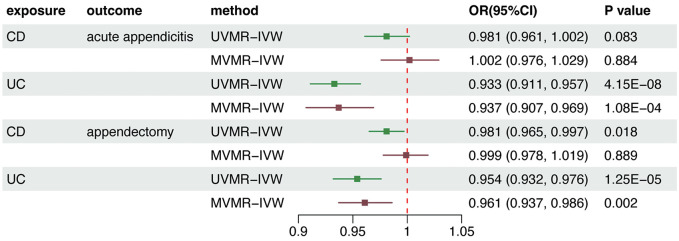
Association between MVMR analyses. **Direct Effect Estimates of genetically predisposed IBD, CD, and UC on acute appendicitis and appendectomy from MVMR.** Estimated odds ratios (ORs) represent the effect of per log-OR increase in IBD, CD, and UC on appendicitis and appendectomy using the IVW method. MR, Mendelian randomization; IBD, inflammatory bowel disease; UC, ulcerative colitis; CD, Crohn’s disease.

In UVMR analyses, the IVW method revealed that genetically predicted IBD significantly decreased the risk of acute appendicitis (OR = 0.954, 95% CI = 0.935–0.974, p = 5.28e-06) and appendectomy (OR = 0.966, 95% CI = 0.948–0.983, p = 1.75e-04) (Fig 3). Subgroup analyses suggested that this effect was mainly attributed to UC. Genetically predicted UC was causally associated with a significantly lower risk of acute appendicitis (OR = 0.933, 95% CI = 0.911–0.957, p = 4.15e-08) and appendectomy (OR = 0.954, 95% CI = 0.932–0.976, p = 1.25e-05). The result was consistent across all other three complementary MR methods (S9-S10 Table). CD has no significant association with acute appendicitis but has a suggestive association with appendectomy (OR = 0.981, 95% CI = 0.965–0.997, p = 0.018) (Fig 4). Considering the overlap of genetic instruments between both CD and UC, we performed MVMR analyses to calculate the direct effects.

The estimates presented for the main results originated from the multivariable robust IVW model with multiplicative random effects. Genetically predicted UC was causally associated with a lower risk of acute appendicitis (OR, 0.937; CI = 0.907–0.957; p < 1.08e-04) and appendectomy (OR, 0.961; CI = 0.937–0.986; p = 0.002) while after excluding the influence of UC, genetically predicted CD was not associated with both acute appendicitis and appendectomy (all p > 0.8) (Fig 4, S5 Fig, and S3 Table). Furthermore, S6 and S7 Figs illustrate the forest plots and leave-one-out analysis of the causal effects of genetically predicted IBD and its subtypes on acute appendicitis and appendectomy. Lastly, S8 Fig illustrates the funnel plots showing the heterogeneity of each IV.

### Genetic correlation between acute appendicitis and IBD

**Fig 5:**
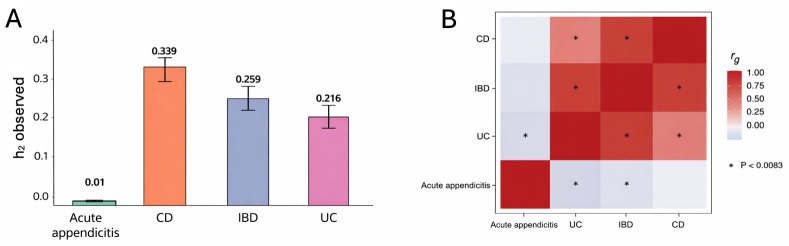
Heritability and Genetic correlation for IBD, CD, UC, and acute appendicitis. **A.** Estimated SNP heritability for each trait in Europeans. The error bar represents sensitivity. **B.** Heat map of genetic correlation (rg). Gray boxes indicate a negative correlation, and red boxes a positive genetic correlation. Genetic correlations that are significant after Bonferroni correction (p < 0.0083) are marked with an asterisk.

The heritability of each trait and the genetic correlation between them are shown in Fig 5. The total heritability of IBD, UC, and CD was 25.88%, 21.55%, and 33.90% respectively (S11-S12 Table). The total heritability of acute appendicitis was 1.04%, which is relatively small. No significant genetic correlation was observed between Crohn’s disease (CD) and acute appendicitis; however, a significant negative genetic correlation was identified between ulcerative colitis (UC) and acute appendicitis (rg = −0.205, p = 0.005) (S13 Table).

## Discussion

Our MR findings newly suggest the association between acute appendicitis, appendectomy, and IBD as well as its main subtypes was elucidated using two-sample bidirectional MR methods. Our bidirectional MR study provides novel genetic evidence that predisposition to UC, associates with reduced risks of acute appendicitis and appendectomy. Notably, this protective relationship does not extend reciprocally from appendicitis to IBD development, challenging previous observational claims of appendectomy’s therapeutic benefits in UC.

Historical observational studies, beginning with a seminal 1987 case-control investigation, consistently reported an inverse association between appendectomy and UC risk [31–34]. This finding gained clinical traction through subsequent replications, with approximately 30 studies suggesting reduced UC incidence post-appendectomy [18,35,36]. However, recent cohort-based analyses and meta-analyses challenge this consensus, demonstrating no significant reduction in UC severity following appendectomy [16]. The persistent discordance across observational studies reflects fundamental methodological constraints: temporal ambiguity in establishing disease onset sequences, susceptibility to selection bias and residual confounding (notably smoking status) [12,37], and an inability to exclude reverse causality wherein subclinical IBD influences appendicitis risk. Our MR approach surmounts these limitations by leveraging genetic variants as instrumental variables, which emulate randomized exposure allocation while minimizing confounding. The resultant causal estimate—genetically predicted UC conferring a protective effect against acute appendicitis (OR = 0.933, 95% CI 0.911–0.957, p < 0.001) and appendectomy (OR = 0.954, 95% CI 0.932–0.976, p < 0.001). This provides robust clarification of the relationship. Crucially, this genetic evidence is previously unresolvable through observational epidemiology.

In the present study, three key insights emerge from our findings. First, the UC-associated genetic architecture may confer biological protection against appendiceal inflammation, supported by significant negative genetic correlation. It may be that the association reported in previous research is driven by genetic confounding, the possible mechanism can be a significant genetic linkage disequilibrium between the susceptibility genes linked to UC and an unidentified genetic trait that confers protection against acute appendicitis. Second, apparent clinical benefits of appendectomy in UC cohorts likely reflect confounding rather than causality. Last, distinct divergence between UC and CD suggests subtype-specific pathogenetic mechanisms. These findings provide genetic evidence that warrants consideration in the ongoing evaluation of clinical observations related to appendectomy in IBD management [12,16]. Importantly, our results are consistent with a recent in vivo study. It has shown that after appendectomy in mice, gut inflammation can be alleviated by inhibiting T cell activation [38], suggesting a potential role of immune regulation in the relationship between appendix and IBD.

The primary strength of this study lies in its application of Mendelian randomization to investigate the bidirectional causal relationships between appendicitis, appendectomy, and IBD, thereby mitigating key limitations of observational studies such as confounding and reverse causation. Our analyses leveraged two independent samples to dissect the associations involving appendicitis and appendectomy separately because a previous epidemiological study reported that appendicitis, rather than appendectomy, was linked to a significantly decreased risk of UC [20]. And we employed multiple sensitivity analyses to assess the robustness of our findings. However, our study also has limitations and weskness. First, we acknowledge that the effect of acute appendicitis or an appendectomy on the risk of IBD may vary depending on whether the condition occurred during childhood or adulthood, as indicated by the findings of some observational studies [6,31,39,40]. However, owing to the lack of an available GWAS dataset on the age of appendectomy, we could not stratify the causal effects of appendectomy on the subsequent risk of IBD based on age. Second, as our data were primarily from individuals of European ancestry, caution is needed when generalizing findings to other populations. Third, while sensitivity analyses suggested no major horizontal pleiotropy, some residual bias from pathways unrelated to IBD cannot be entirely excluded, such as vitamin metabolism [41]. Fourth, our estimates likely reflect population-level averages which may mask stronger effects in specific patient subgroups. Despite MR design strengths, residual pleiotropy or unmeasured confounding cannot be fully excluded. The modest effects may arise from complex, indirect biological mechanisms rather than a single strong pathway, underscoring the need for further investigation to clarify clinical relevance. Last, although the P value demonstrates statistical significance, the ORs are near 1.0, suggesting a relatively modest effect. Our estimates are likely to represent population-level averages, which might obscure more pronounced effects within specific patient subgroups. Despite the inherent strengths of the MR design, it is not possible to completely rule out the presence of residual pleiotropy or unmeasured confounding factors. The observed modest effects may stem from intricate, indirect biological mechanisms rather than a single, dominant pathway. This highlights the imperative for further investigation to elucidate the clinical relevance of these findings.

Our results do not support the hypothesis that the often-observed association between acute appendicitis, appendectomy, and UC is directly causal. In contrast, we found that acute appendicitis and appendectomy have no effect on the risk of UC. Furthermore, reverse UVMR analysis revealed a significant inverse association between UC and appendicitis/appendectomy. The result suggested that the previously observed inverse association between acute appendicitis, appendectomy, and UC is consistent with directionality from UC liability and shared genetic architecture. At present, no randomized clinical trial data have been published to verify the effect of appendectomy on the clinical course of IBD; nevertheless, a randomized external pilot trial protocol that aimed to examine the efficacy of appendectomy to maintain remission among a patient with UC has been published [42]. Based on the findings of the present MR analysis, we recommend additional longitudinal studies with meticulous designs and rigorous conduction to determine the directionality of this causal association. In addition, nvestigating the genetic mechanisms behind the inverse relationship between appendicitis and IBD could be an important future direction. CRP-QTL co-mapping analysis could be used to identify genetic loci shared between appendicitis and IBD, such as STAT3 and IL2RA, and organoid models were used to validate their functions.

## Conclusion

In summary, our bidirectional MR analysis reveals novel evidence that UC may confer a protective effect against acute appendicitis development, while refuting a causal role of appendectomy in reducing IBD incidence. These findings suggest that previously observed clinical associations between appendectomy and UC likely reflect reverse causation and genetic confounding rather than direct biological effects. The study underscores the appendix as a potential immunomodulatory organ in UC pathogenesis, warranting investigation into shared genetic mechanisms. Future research should prioritize longitudinal cohorts to validate these causal inferences and elucidate clinical implications. We advise caution in considering appendix-targeted interventions for IBD management until mechanistic studies clarify this complex relationship.

## Supporting information

S1 Fig MR scatter plot for appendicitis and appendectomy on IBD, CD, and UC. (DOCX)

S2 Fig MR forest plot for appendicitis and appendectomy on IBD, CD, and UC. (DOCX)

S3 Fig MR leave-one-out plot for appendicitis and appendectomy on IBD, CD, and UC. (DOCX)

S4 Fig MR funnel plot for appendicitis and appendectomy on IBD, CD, and UC. (DOCX)

S5 Fig MR scatter plot for IBD, CD, and UC on appendicitis and appendectomy. (DOCX)

S6 Fig MR forest plot for IBD, CD, and UC on appendicitis and appendectomy. (DOCX)

S7 Fig MR leave-one-out plot for IBD, CD, and UC on appendicitis and appendectomy. (DOCX)

S8 Fig MR funnel plot for IBD on appendicitis and appendectomy. (DOCX)

S1 Table Summary of Genetic Instruments identified for MR Analyses. (DOCX)

S2 Table Genetic variants used as instrumental variables for acute appendicitis. (DOCX)

S3 Table Genetic variants used as instrumental variables for appendectomy. (DOCX)

S4 Table Heterogeneity and pleiotropy analyses of appendicitis and appendectomy on IBD and it’s subtypes. (DOCX)

S5 Table Univariable MR estimates from different methods of assessing the causal effect of appendicitis and appendectomy on IBD and its subtypes. (DOCX)

S6 Table Genetic variants used as instrumental variables for IBD. (DOCX)

S7 Table Genetic variants used as instrumental variables for CD. (DOCX)

S8 Table Genetic variants used as instrumental variables for UC. (DOCX)

S9 Table Univariable MR estimates from different methods of assessing the causal effect of IBD and its subtypes on appendicitis and appendectomy. (DOCX)

S10 Table Multivariable MR estimates for UC and CD using IVW method. (DOCX)

S11 Table Heterogeneity and pleiotropy analyses of IBD and it’s subtypes with appendicitis and appendectomy. (DOCX)

S12 Table LDSC Regression Estimates of IBD, UC, CD, and appendicitis. (DOCX)

S13 Table Genetic Correlation Estimates between IBD and appendicitis from LDSC Regression. (DOCX)
