# Supplementary figures and images for: Evidence for genetic correlation between appendix and inflammatory bowel disease: A bidirectional Mendelian randomization study
Source: PLoS One. 2026 Feb 11;21(2):e0342541. doi: 10.1371/journal.pone.0342541 (PMC12893558; doi:10.1371/journal.pone.0342541)

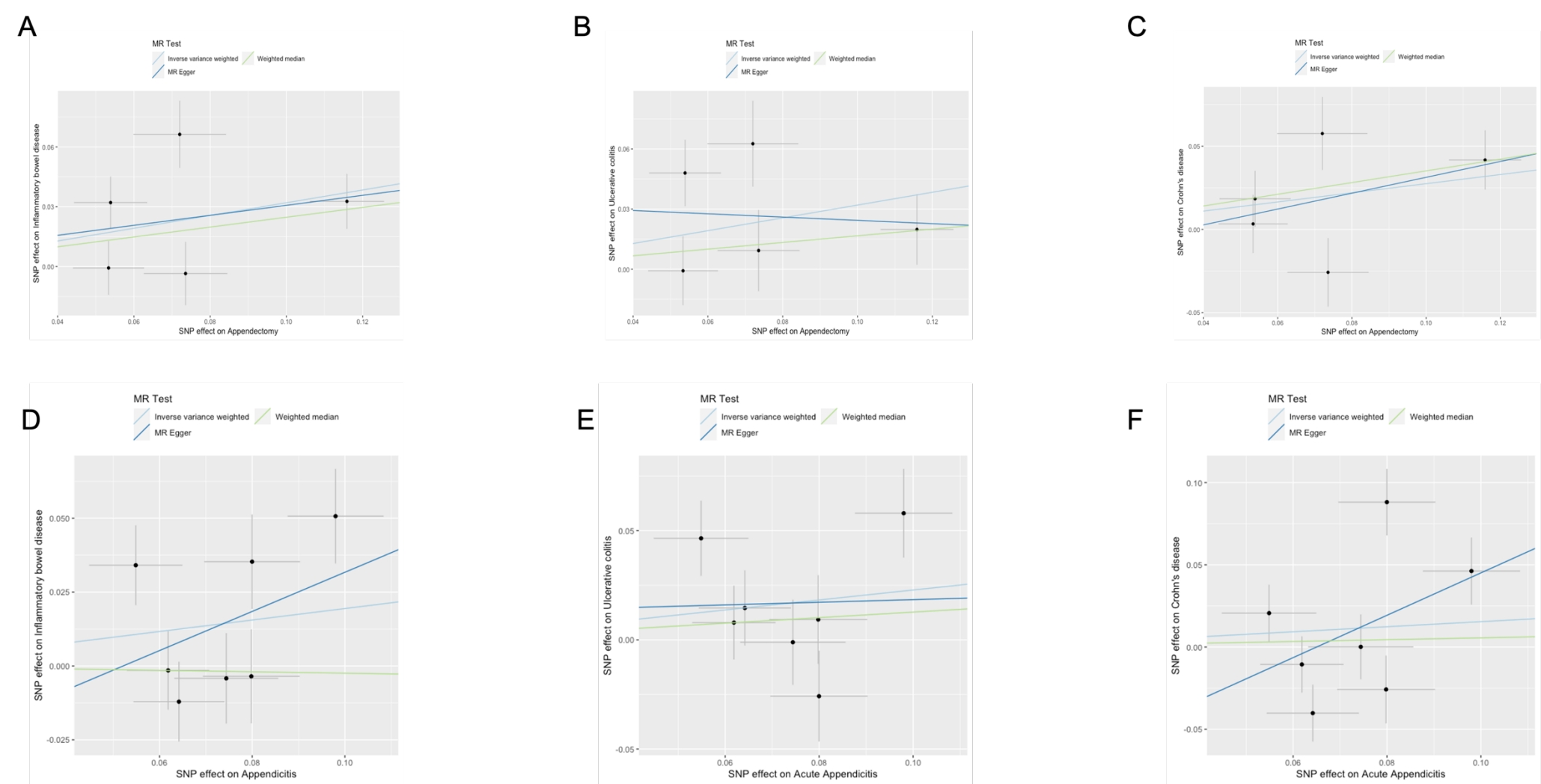


**Figure S1** MR scatter plot for appendicitis and appendectomy on IBD, CD, and UC.

Supplement: S1 Fig — (DOCX) [file pone.0342541.s001.docx]

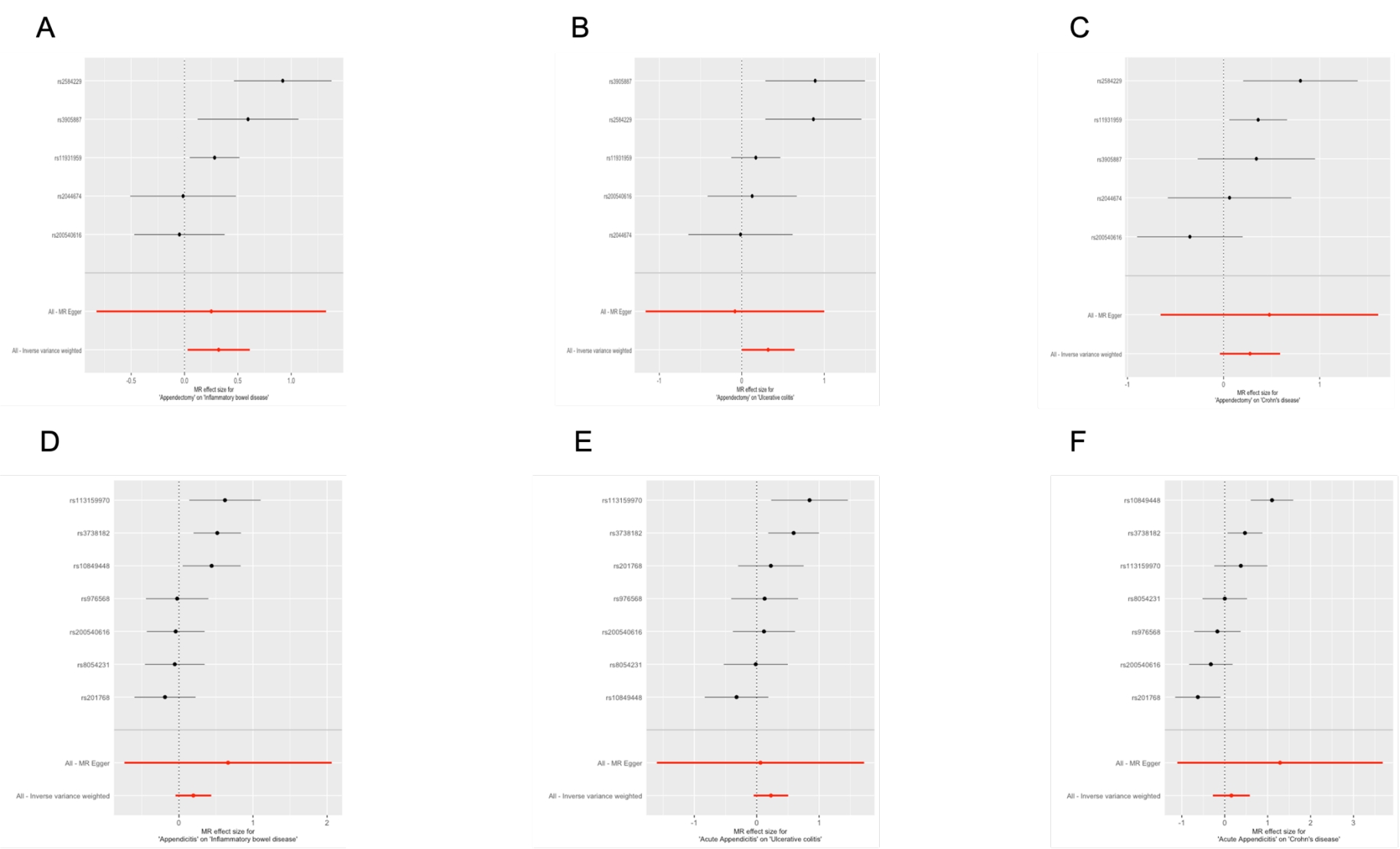


**Figure S2** MR forest plot for appendicitis and appendectomy on IBD, CD, and UC.

Supplement: S2 Fig — (DOCX) [file pone.0342541.s002.docx]

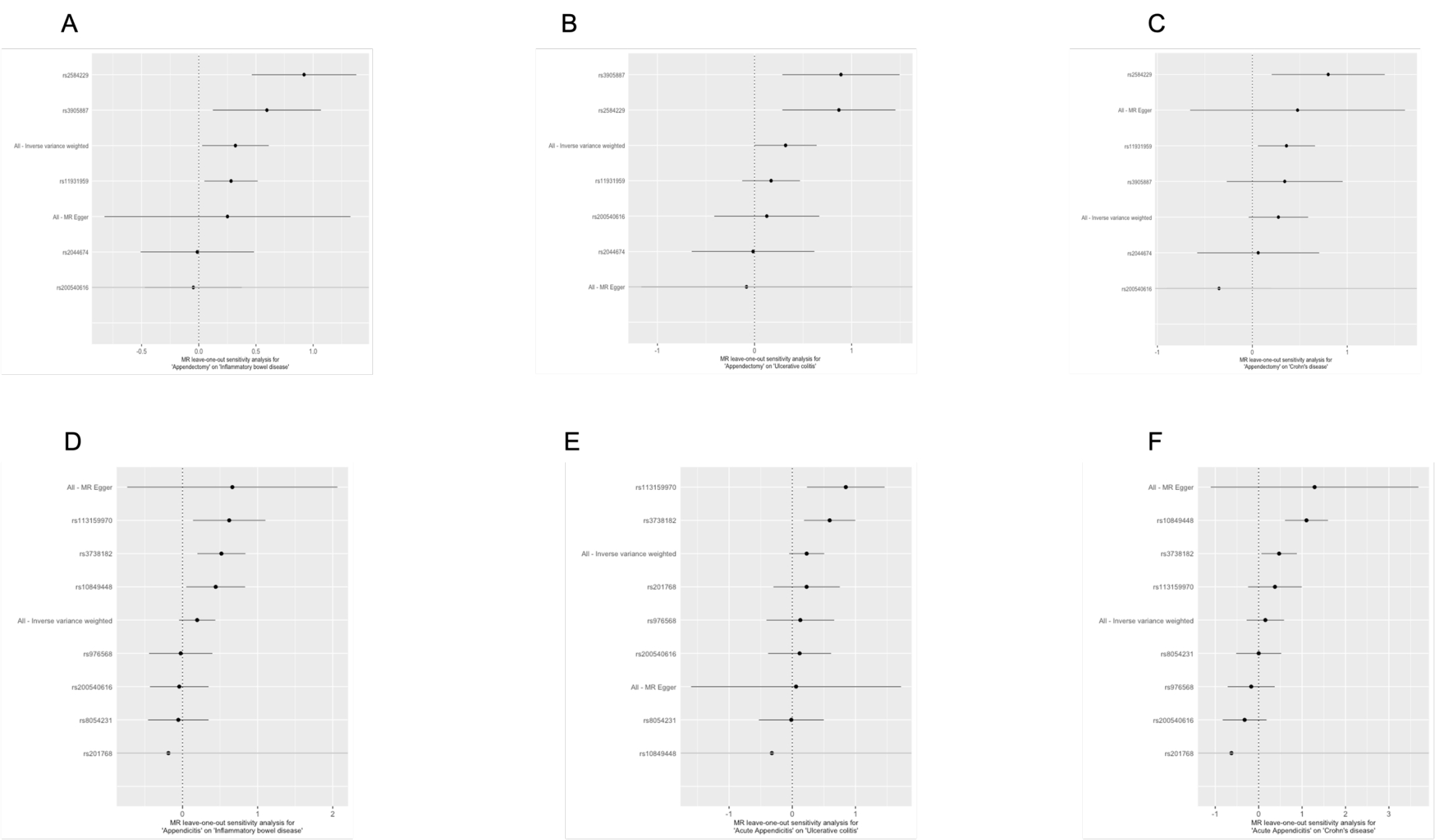


**Figure S3** MR leave-one-out plot for appendicitis and appendectomy on IBD, CD, and UC.

Supplement: S3 Fig — (DOCX) [file pone.0342541.s003.docx]

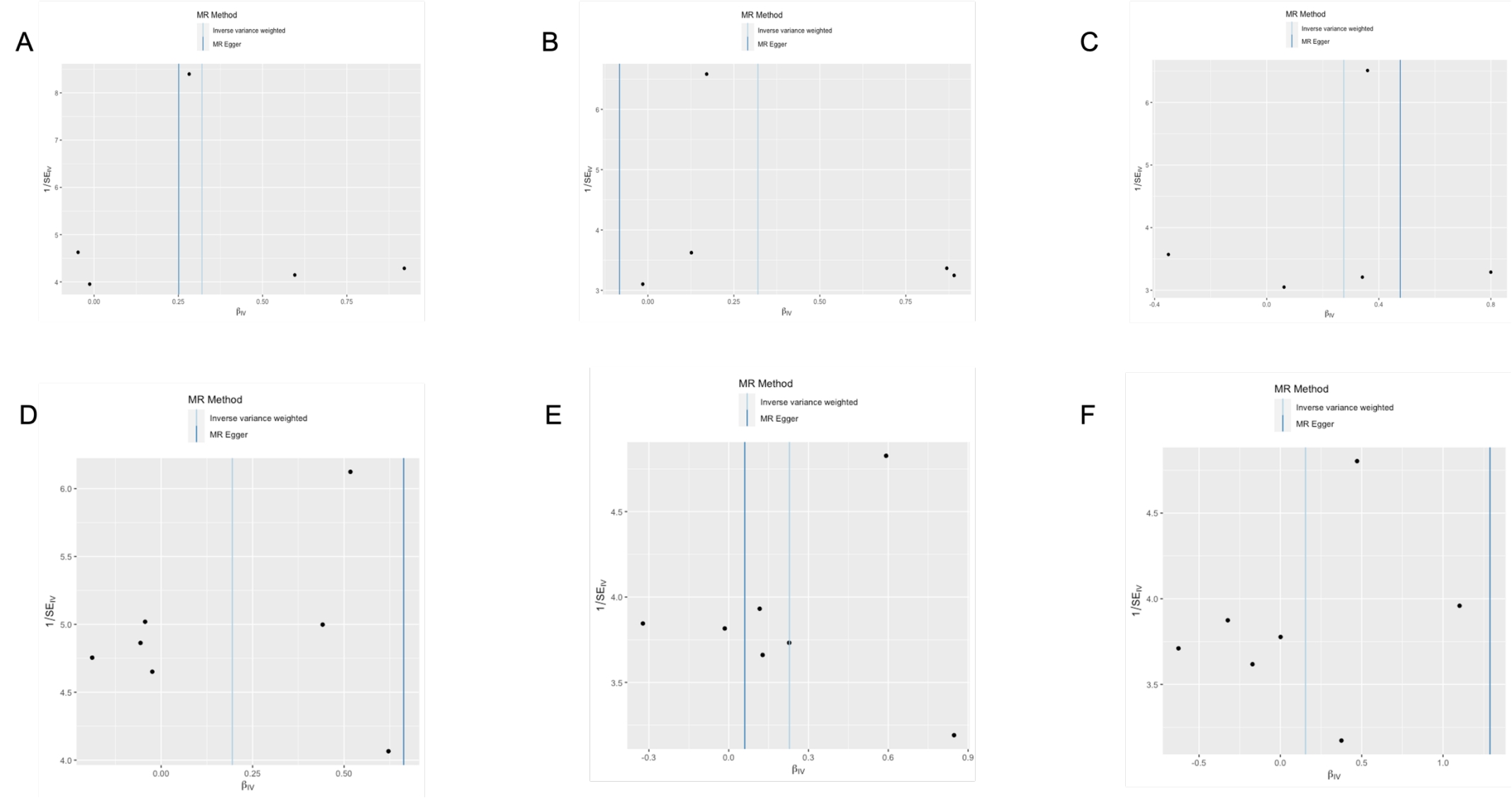


**Figure S4** MR funnel plot for appendicitis and appendectomy on IBD, CD, and UC.

Supplement: S4 Fig — (DOCX) [file pone.0342541.s004.docx]

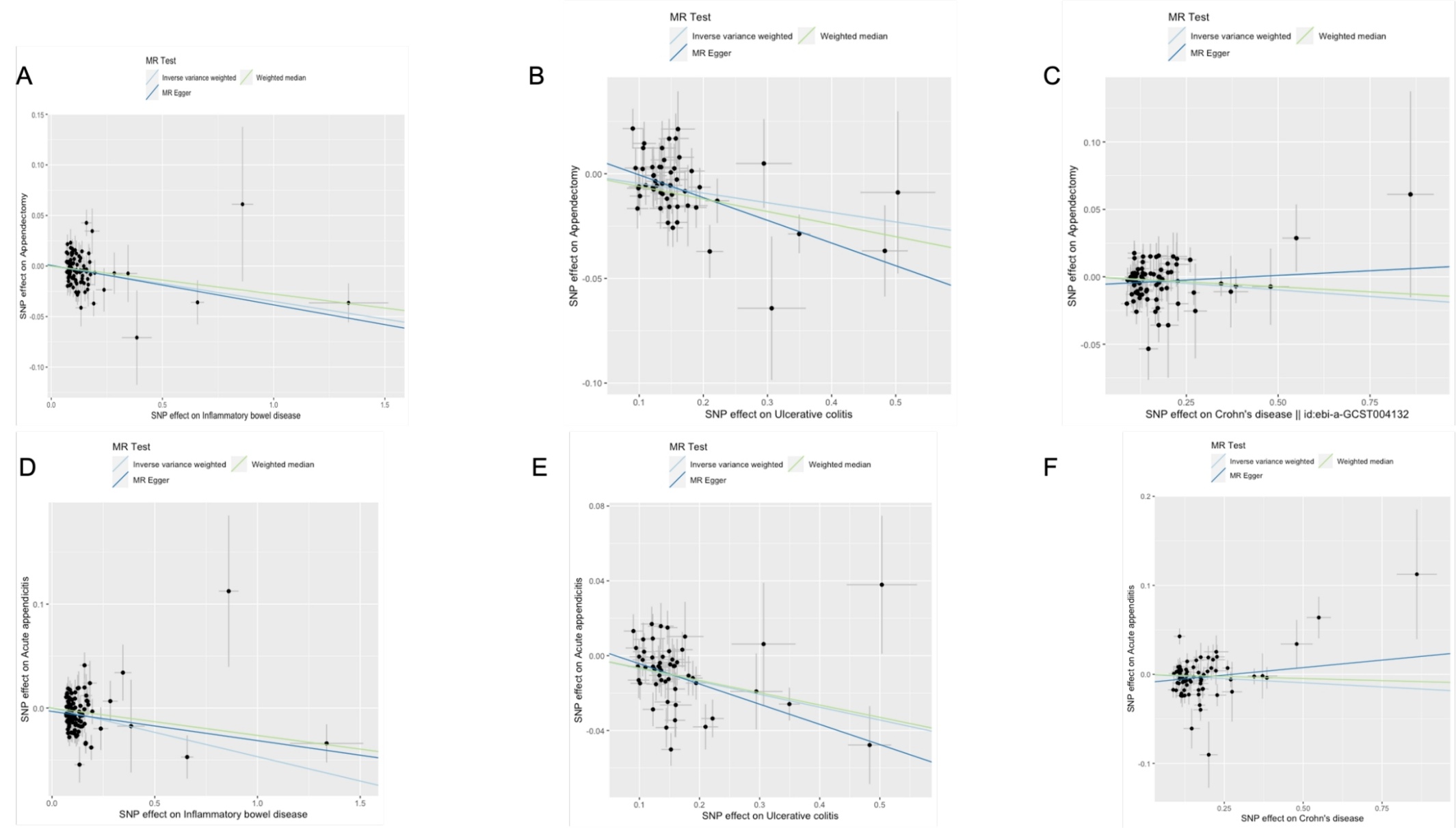


**Figure S5** MR scatter plot for IBD, CD, and UC on appendicitis and appendectomy.

Supplement: S5 Fig — (DOCX) [file pone.0342541.s005.docx]

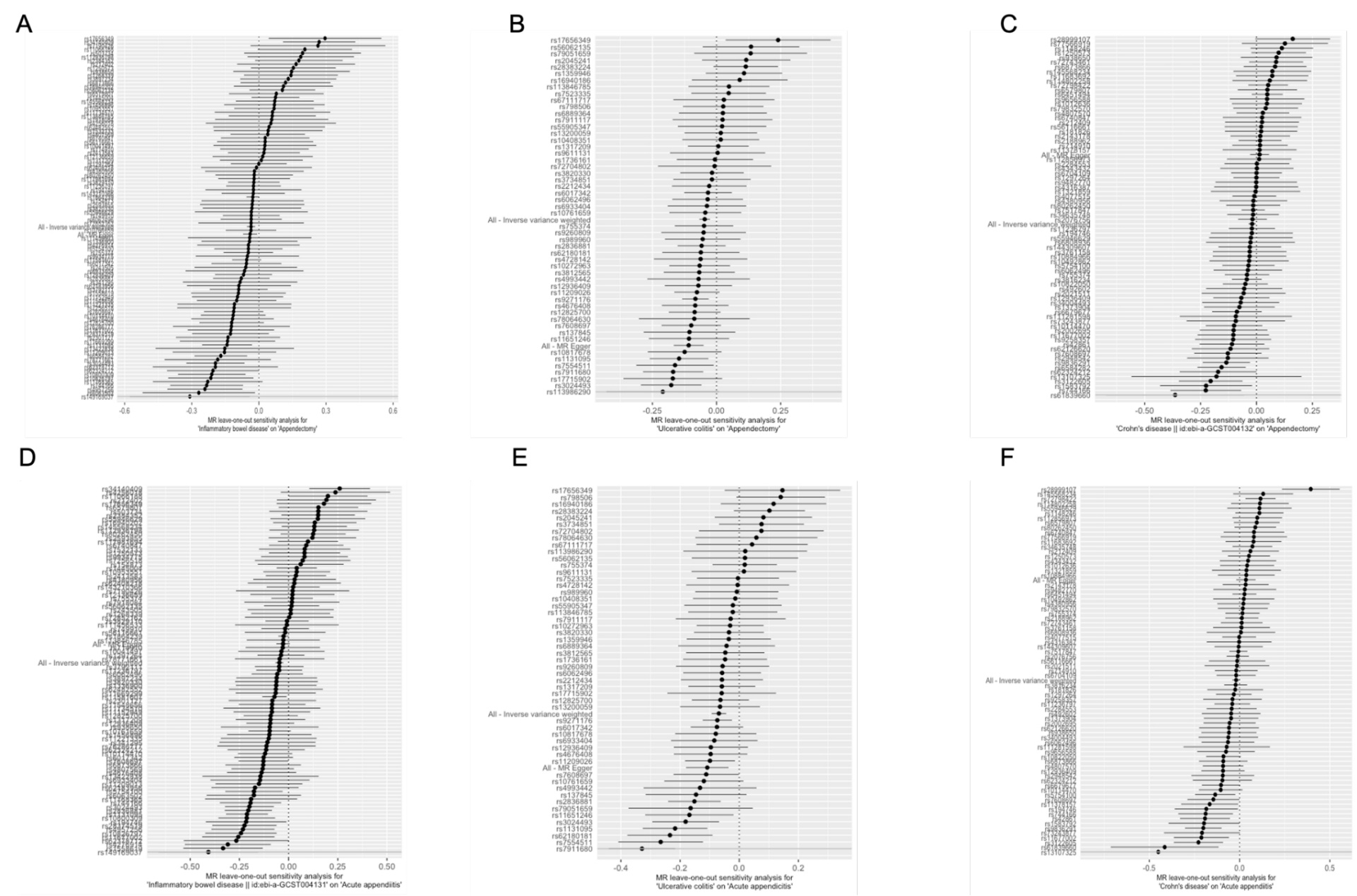


**Figure S6** MR forest plot for IBD, CD, and UC on appendicitis and appendectomy.

Supplement: S6 Fig — (DOCX) [file pone.0342541.s006.docx]

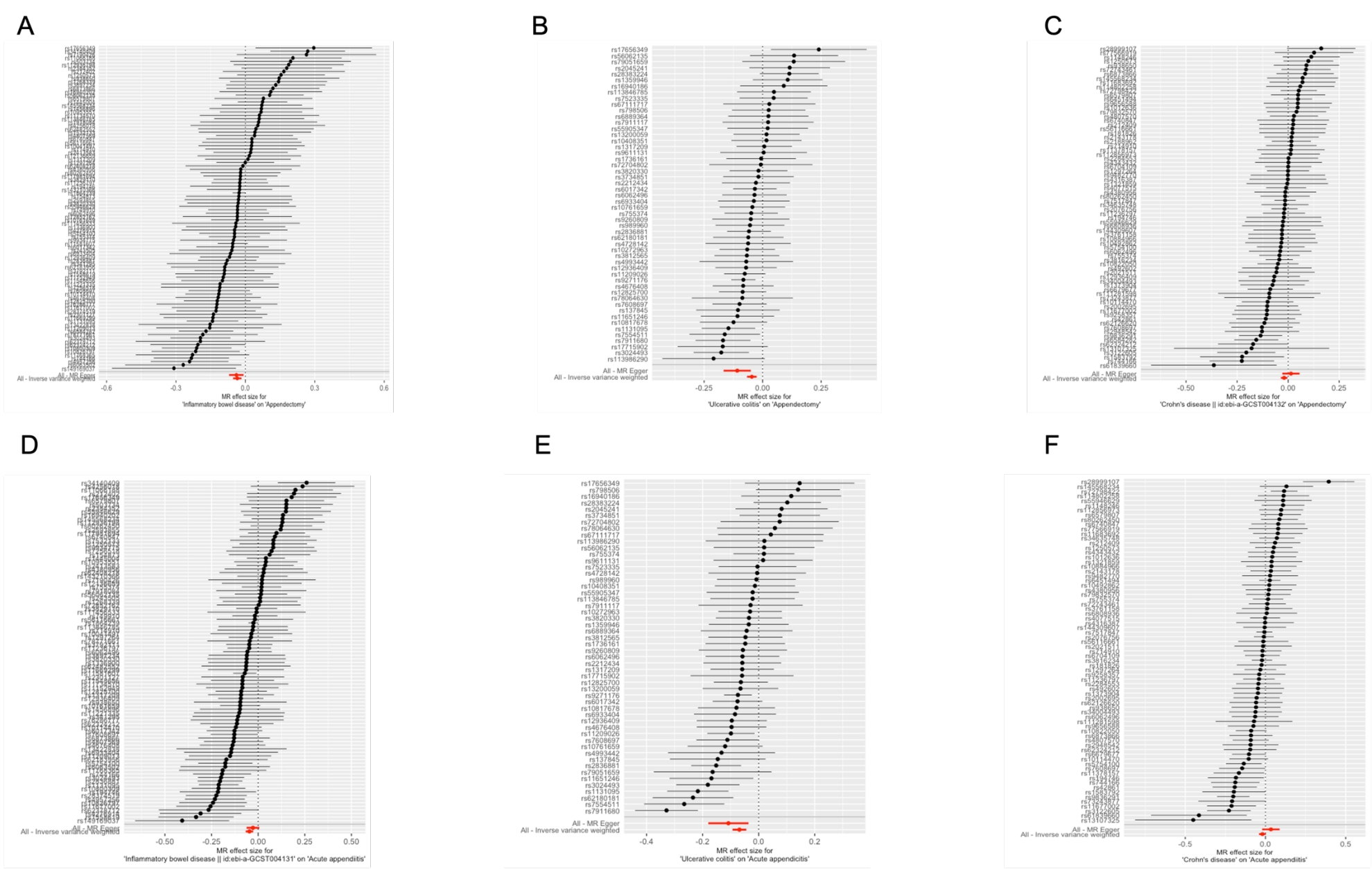


**Figure S7** MR leave-one-out plot for IBD, CD, and UC on appendicitis and appendectomy.

Supplement: S7 Fig — (DOCX) [file pone.0342541.s007.docx]

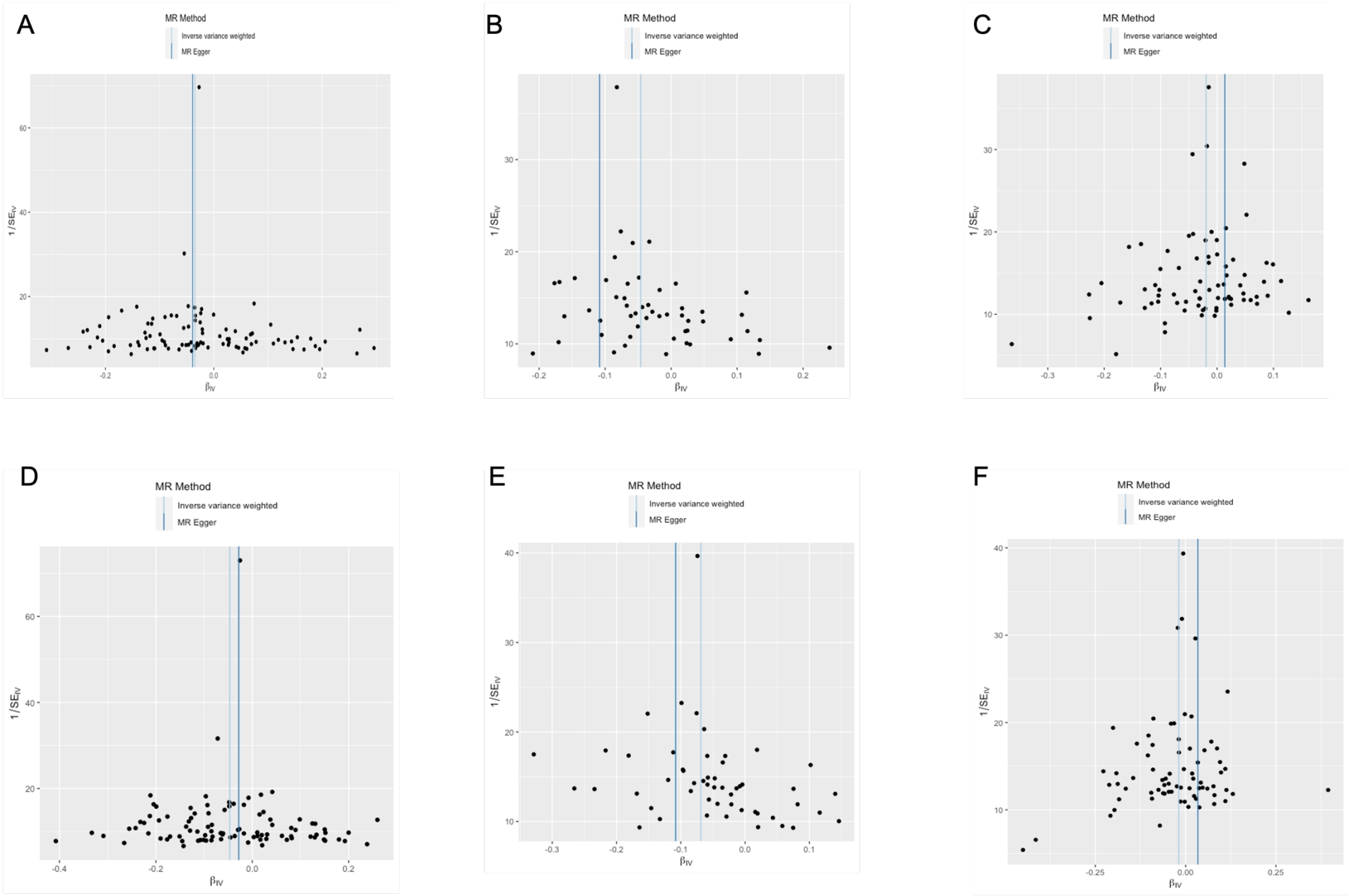


**Figure S8** MR funnel plot for IBD on appendicitis and appendectomy.

Supplement: S8 Fig — (DOCX) [file pone.0342541.s008.docx]
