# Supplementary material for: Evidence for genetic correlation between appendix and inflammatory bowel disease: A bidirectional Mendelian randomization study
Source: PLoS One. 2026 Feb 11;21(2):e0342541. doi: 10.1371/journal.pone.0342541 (PMC12893558; doi:10.1371/journal.pone.0342541)
Supplement: S1 Table — (DOCX) [file pone.0342541.s009.docx]

**Table S1: Summary of Genetic Instruments identified for MR Analyses**

| **Exposure** | **Significant level** | **No. of SNPs** | **F statistics*** |
| --- | --- | --- | --- |
| **Appendicitis** | 5e-08 | 12 | 668.9 (429.7 –4584.5) |
| **Appendectomy** | 5e-08 | 7 | 526.5 (501.8 –2170.7) |
| **IBD** | | | |
| IBD | 5e-08 | 117 | 241.0(87.5 –4254.1) |
| CD | 5e-08 | 89 | 270.8 (112.7 – 2474.9) |
| UC | 5e-08 | 62 | 325.1 (174.8 –2595.0) |

^*^ Median and range (minimum and maximum).

IBD: Inflammatory Bowel Disease; CD: Crohn’s Disease; UC: Ulcerative Colitis.
