# Supplementary material for: Evidence for genetic correlation between appendix and inflammatory bowel disease: A bidirectional Mendelian randomization study
Source: PLoS One. 2026 Feb 11;21(2):e0342541. doi: 10.1371/journal.pone.0342541 (PMC12893558; doi:10.1371/journal.pone.0342541)
Supplement: S2 Table — (DOCX) [file pone.0342541.s010.docx]

**Table S2: genetic variants used as instrumental variables for appendicitis.**

| SNP | other allele | Effect allele | eaf | se | beta | pval | R2 | F |
| --- | --- | --- | --- | --- | --- | --- | --- | --- |
| rs3738182 | G | A | 0.239914 | 0.0103513 | -0.0979872 | 2.90E-21 | 0.00350177 | 1317.86784 |
| rs1550771 | T | C | 0.795866 | 0.0106842 | -0.0624286 | 5.12E-09 | 0.00126635 | 475.514953 |
| rs200540616 | C | T | 0.216786 | 0.0104351 | 0.079808 | 2.04E-14 | 0.00216289 | 812.898367 |
| rs976568 | G | T | 0.599276 | 0.00887135 | -0.0618654 | 3.09E-12 | 0.00183822 | 690.650495 |
| rs7697491 | A | T | 0.530804 | 0.00868103 | -0.155711 | 6.07E-72 | 0.01207694 | 4584.53538 |
| rs2348650 | C | G | 0.658643 | 0.009235 | 0.0716928 | 8.28E-15 | 0.00231121 | 868.77289 |
| rs9273368 | G | A | 0.278145 | 0.0096078 | 0.0644853 | 1.92E-11 | 0.00166983 | 627.277708 |
| rs10748784 | G | A | 0.542511 | 0.00875832 | 0.0512173 | 4.98E-09 | 0.00130212 | 488.967296 |
| rs10849448 | A | G | 0.756301 | 0.0103436 | 0.0799734 | 1.06E-14 | 0.0023576 | 886.249525 |
| rs201768 | T | C | 0.724155 | 0.00980694 | 0.0641906 | 5.93E-11 | 0.00164615 | 618.367131 |
| rs113159970 | G | A | 0.255019 | 0.0100646 | -0.0548815 | 4.96E-08 | 0.00114446 | 429.693091 |
| rs8054231 | G | A | 0.192681 | 0.0112047 | -0.0744085 | 3.12E-11 | 0.0017225 | 647.096804 |
