# Supplementary material for: Evidence for genetic correlation between appendix and inflammatory bowel disease: A bidirectional Mendelian randomization study
Source: PLoS One. 2026 Feb 11;21(2):e0342541. doi: 10.1371/journal.pone.0342541 (PMC12893558; doi:10.1371/journal.pone.0342541)
Supplement: S3 Table — (DOCX) [file pone.0342541.s011.docx]

**Table S3: genetic variants used as instrumental variables for appendectomy.**

| SNP | other allele | effect allele | eaf | se | beta | pval | R2 | F |
| --- | --- | --- | --- | --- | --- | --- | --- | --- |
| rs2484697 | A | G | 0.497538 | 0.00912702 | 0.054711 | 2.04E-09 | 0.00149661 | 526.490339 |
| rs200540616 | C | T | 0.21676 | 0.0109459 | 0.0735558 | 1.82E-11 | 0.00183712 | 646.498615 |
| rs2044674 | C | T | 0.389458 | 0.00932122 | 0.0533642 | 1.03E-08 | 0.00135427 | 476.349702 |
| rs11931959 | A | G | 0.307617 | 0.00973014 | 0.115885 | 1.05E-32 | 0.00572059 | 2020.98877 |
| rs3905887 | C | T | 0.646222 | 0.00958465 | 0.0539003 | 1.87E-08 | 0.00132839 | 467.232869 |
| rs7983949 | T | A | 0.267565 | 0.010399 | 0.0586683 | 1.68E-08 | 0.00134907 | 474.51818 |
| rs2584229 | T | C | 0.17522 | 0.0121503 | -0.072019 | 3.08E-09 | 0.00149915 | 527.386124 |
