# Supplementary material for: Evidence for genetic correlation between appendix and inflammatory bowel disease: A bidirectional Mendelian randomization study
Source: PLoS One. 2026 Feb 11;21(2):e0342541. doi: 10.1371/journal.pone.0342541 (PMC12893558; doi:10.1371/journal.pone.0342541)
Supplement: S4 Table — (DOCX) [file pone.0342541.s012.docx]

**Table S4: Heterogeneity and pleiotropy analyses of appendicitis and appendectomy on IBD and it’s subtypes.**

| Exposure | Outcome | MR Egger | | | | IVW | |
| --- | --- | --- | --- | --- | --- | --- | --- |
|  |  | Intercept | Pleiotropy  p-value | Cochran’s Q  statistic | Heterogeneity  p-value | Cochran’s Q  statistic | Heterogeneity  p-value |
| Appendicitis | IBD | -0.035 | 0.534 | 14.432 | 0.013 | 15.717 | 0.015 |
|  | UC | 0.012 | 0.848 | 12.555 | 0.028 | 12.6570 | 0.049 |
|  | CD | -0.084 | 0.388 | 25.805 | <0.001 | 30.412 | <0.001 |
| Appendectomy | IBD | 0.006 | 0.903 | 12.586 | 0.006 | 12.660 | 0.013 |
|  | UC | 0.033 | 0.499 | 7.847 | 0.049 | 9.385 | 0.052 |
|  | CD | -0.016 | 0.738 | 8.367 | 0.039 | 8.744 | 0.068 |

IBD: Inflammatory Bowel Disease; CD: Crohn’s Disease; UC: Ulcerative Colitis, IVW: Inverse variance weighted.
