# Supplementary material for: Evidence for genetic correlation between appendix and inflammatory bowel disease: A bidirectional Mendelian randomization study
Source: PLoS One. 2026 Feb 11;21(2):e0342541. doi: 10.1371/journal.pone.0342541 (PMC12893558; doi:10.1371/journal.pone.0342541)
Supplement: S5 Table — (DOCX) [file pone.0342541.s013.docx]

**Table S5: MR estimates from different methods of assessing the causal effect of appendicitis and appendectomy on IBD and its subtypes.**

| Exposure | Outcome | No. of SNP | IVW | | WM | | MR-Egger | | MR PRESSO (outlier-corrected) | | |
| --- | --- | --- | --- | --- | --- | --- | --- | --- | --- | --- | --- |
|  |  |  | OR (95%CI) | P value | OR (95%CI) | P value | OR (95% CI) | P value | OR | P | P (global test) |
| Appendicitis | IBD | 7 | 1.215 (0.954, 1.547) | 0.115 | 0.976 (0.759, 1.254) | 0.848 | 1.941 (0.479, 7.864) | 0.396 | NA | NA | 0.024 |
|  | UC | 7 | 1.256(0.952, 1.658) | 0.106 | 1.136(0.851, 1.517) | 0.388 | 1.062 (0.202, 5.592) | 0.946 | NA | NA | 0.066 |
|  | CD | 7 | 1.167 (0.759, 1.796) | 0.482 | 1.057 (0.765, 1.463) | 0.735 | 3.627 (0.332, 39.671) | 0.339 | 1.105 | 0.565 | <0.001 |
| Appendectomy | IBD | 5 | 1.377 (1.029, 1.844) | 0.031 | 1.281 (1.044, 1.570) | 0.017 | 1.285 (0.438, 3.767) | 0.678 | NA | NA | 0.066 |
|  | UC | 5 | 1.377(1.000, 1.897) | 0.050 | 1.181(0.910, 1.533) | 0.210 | 0.921 (0.312, 2.720) | 0.891 | NA | NA | 0.154 |
|  | CD | 5 | 1.316 (0.962, 1.801) | 0.086 | 1.421 (1.085, 1.862) | 0.011 | 1.611 (0.519, 5.000) | 0.470 | NA | NA | 0.154 |

IVW: Inverse variance weighted; WM: Weighted median; MR PRESSO: MR Pleiotropy RESidual Sum and Outlier; NA: not available; OR: Odds Ratio; CI: Confidence Interval; IBD: Inflammatory Bowel Disease; CD: Crohn’s Disease; UC: Ulcerative Colitis.
