# Supplementary material for: Evidence for genetic correlation between appendix and inflammatory bowel disease: A bidirectional Mendelian randomization study
Source: PLoS One. 2026 Feb 11;21(2):e0342541. doi: 10.1371/journal.pone.0342541 (PMC12893558; doi:10.1371/journal.pone.0342541)
Supplement: S6 Table — (DOCX) [file pone.0342541.s014.docx]

**Table S6: genetic variants used as instrumental variables for IBD.**

| SNP | other allele | effect_allele | eaf | se | beta | pval | R2 | F |
| --- | --- | --- | --- | --- | --- | --- | --- | --- |
| rs12136659 | T | C | 0.2515 | 0.0142 | 0.087 | 1.02E-09 | 0.00284969 | 171.341702 |
| rs2488398 | G | C | 0.1899 | 0.0149 | 0.0985 | 3.63E-11 | 0.00298515 | 179.510491 |
| rs10746475 | T | A | 0.83 | 0.0164 | 0.1308 | 1.58E-15 | 0.00482806 | 290.87057 |
| rs4654925 | G | C | 0.4443 | 0.0124 | -0.1172 | 4.80E-21 | 0.00678269 | 409.433201 |
| rs112936798 | A | C | 0.0219 | 0.0332 | -0.1844 | 2.89E-08 | 0.00145673 | 87.465689 |
| rs35730213 | G | C | 0.2744 | 0.014 | -0.1346 | 7.50E-22 | 0.00721442 | 435.683832 |
| rs3024493 | C | A | 0.166 | 0.0165 | 0.1911 | 4.04E-31 | 0.01011173 | 612.441679 |
| rs11209013 | A | G | 0.5249 | 0.0124 | 0.0773 | 4.46E-10 | 0.00298024 | 179.214121 |
| rs11581607 | G | A | 0.0616 | 0.0294 | -0.6578 | 4.59E-111 | 0.05002493 | 3157.18217 |
| rs1336900 | G | A | 0.4095 | 0.0128 | -0.0848 | 2.98E-11 | 0.00347773 | 209.234794 |
| rs10800309 | A | G | 0.6998 | 0.0133 | -0.123 | 1.94E-20 | 0.0063566 | 383.54798 |
| rs1268339 | T | C | 0.2167 | 0.0163 | 0.0907 | 2.75E-08 | 0.00279275 | 167.908077 |
| rs1317209 | G | A | 0.1809 | 0.016 | 0.1164 | 3.79E-13 | 0.00401524 | 241.704181 |
| rs3820330 | C | A | 0.3002 | 0.014 | -0.0892 | 1.72E-10 | 0.00334306 | 201.105544 |
| rs4276914 | G | A | 0.494 | 0.0125 | 0.0783 | 3.15E-10 | 0.003065 | 184.327253 |
| rs7532133 | A | G | 0.6769 | 0.0134 | 0.0789 | 3.83E-09 | 0.00272299 | 163.70241 |
| rs11677002 | T | C | 0.4592 | 0.0126 | -0.0931 | 1.37E-13 | 0.00430495 | 259.219088 |
| rs55946629 | C | A | 0.1233 | 0.018 | 0.1298 | 5.45E-13 | 0.00364245 | 219.181379 |
| rs4676408 | G | A | 0.5119 | 0.013 | 0.1011 | 7.63E-15 | 0.00510771 | 307.80494 |
| rs7608697 | A | C | 0.3628 | 0.0126 | 0.1395 | 1.67E-28 | 0.00899749 | 544.342333 |
| rs13422838 | T | C | 0.0954 | 0.0205 | -0.1143 | 2.56E-08 | 0.0022549 | 135.498104 |
| rs62180107 | G | C | 0.3688 | 0.0132 | -0.0797 | 1.55E-09 | 0.00295736 | 177.834582 |
| rs3792111 | C | T | 0.5368 | 0.0124 | 0.1391 | 5.12E-29 | 0.009622 | 582.491693 |
| rs1558619 | G | T | 0.493 | 0.0123 | -0.0843 | 8.90E-12 | 0.00355255 | 213.752415 |
| rs76286777 | T | C | 0.2048 | 0.0151 | 0.0996 | 4.65E-11 | 0.00323113 | 194.350624 |
| rs72852162 | A | C | 0.1103 | 0.0202 | -0.1129 | 2.30E-08 | 0.00250171 | 150.366202 |
| rs6740847 | A | G | 0.5447 | 0.0125 | -0.0924 | 1.22E-13 | 0.00423476 | 254.974888 |
| rs62183956 | C | T | 0.5 | 0.0125 | -0.078 | 4.49E-10 | 0.003042 | 182.939612 |
| rs1131095 | T | C | 0.3241 | 0.0131 | 0.1635 | 1.22E-35 | 0.01171189 | 710.507758 |
| rs56116661 | C | T | 0.1938 | 0.0163 | -0.1 | 9.27E-10 | 0.00312483 | 187.936525 |
| rs77272631 | G | C | 0.0408 | 0.0417 | 0.2293 | 3.72E-08 | 0.00411536 | 247.755788 |
| rs503734 | A | G | 0.5139 | 0.0124 | -0.0692 | 2.67E-08 | 0.00239247 | 143.784513 |
| rs2593855 | C | T | 0.325 | 0.014 | -0.0832 | 2.54E-09 | 0.00303713 | 182.646017 |
| rs11734570 | G | A | 0.4473 | 0.0127 | 0.0694 | 4.80E-08 | 0.00238143 | 143.119293 |
| rs62324212 | C | A | 0.4642 | 0.0127 | 0.0886 | 2.67E-12 | 0.00390486 | 235.033555 |
| rs4957256 | C | T | 0.2326 | 0.0155 | -0.1179 | 3.37E-14 | 0.00496237 | 299.002633 |
| rs17656349 | C | T | 0.5646 | 0.0125 | 0.0731 | 5.17E-09 | 0.00262721 | 157.929019 |
| rs6579807 | C | T | 0.16 | 0.0189 | 0.125 | 4.01E-11 | 0.0042 | 252.873067 |
| rs1445004 | C | T | 0.5895 | 0.0127 | 0.1689 | 3.48E-40 | 0.01380659 | 839.362524 |
| rs62378712 | T | C | 0.2634 | 0.0142 | -0.0776 | 4.23E-08 | 0.00233669 | 140.42432 |
| rs6873866 | T | C | 0.5954 | 0.0128 | -0.0919 | 6.15E-13 | 0.00406908 | 244.958163 |
| rs10041497 | T | C | 0.6203 | 0.0129 | 0.0819 | 1.95E-10 | 0.00315966 | 190.037794 |
| rs755374 | C | T | 0.327 | 0.0134 | 0.1767 | 1.59E-39 | 0.01374251 | 835.412564 |
| rs56235845 | T | G | 0.341 | 0.0138 | 0.0877 | 1.77E-10 | 0.00345676 | 207.968824 |
| rs11739135 | G | C | 0.3738 | 0.0125 | 0.1366 | 1.10E-27 | 0.00873542 | 528.347399 |
| rs341295 | C | T | 0.4652 | 0.0124 | 0.0702 | 1.45E-08 | 0.00245208 | 147.376068 |
| rs11152949 | A | G | 0.3032 | 0.0133 | 0.1019 | 1.56E-14 | 0.00438749 | 264.21092 |
| rs1267496 | G | C | 0.7694 | 0.0159 | 0.1053 | 3.39E-11 | 0.00393458 | 236.829483 |
| rs145568234 | T | G | 0.0139 | 0.0476 | 0.86 | 4.73E-73 | 0.02027508 | 1240.74894 |
| rs6457681 | G | T | 0.2197 | 0.0153 | -0.1687 | 3.75E-28 | 0.0097578 | 590.793626 |
| rs4712528 | G | C | 0.7932 | 0.0152 | 0.1043 | 7.14E-12 | 0.00356888 | 214.738531 |
| rs143210366 | T | G | 0.0398 | 0.036 | 0.2836 | 3.14E-15 | 0.00614734 | 370.843461 |
| rs62408218 | C | T | 0.3757 | 0.0129 | -0.0818 | 2.40E-10 | 0.00313885 | 188.78256 |
| rs212402 | G | A | 0.6431 | 0.013 | -0.0743 | 1.06E-08 | 0.00253415 | 152.321095 |
| rs34140409 | C | T | 0.1272 | 0.0237 | -0.1583 | 2.28E-11 | 0.00556408 | 335.461188 |
| rs6933404 | T | C | 0.169 | 0.0149 | 0.0863 | 6.64E-09 | 0.00209189 | 125.682315 |
| rs35171809 | A | G | 0.4473 | 0.0123 | 0.1088 | 1.16E-18 | 0.00585297 | 352.980681 |
| rs10953551 | A | G | 0.6034 | 0.0127 | -0.1033 | 4.94E-16 | 0.00510727 | 307.778163 |
| rs243505 | A | G | 0.4185 | 0.0128 | -0.0805 | 3.04E-10 | 0.00315404 | 189.698672 |
| rs149169037 | G | A | 0.0755 | 0.0242 | -0.1338 | 3.26E-08 | 0.00249917 | 150.213246 |
| rs1456896 | C | T | 0.6869 | 0.0133 | 0.0879 | 4.50E-11 | 0.00332341 | 199.919649 |
| rs62482552 | G | A | 0.5726 | 0.0131 | -0.0737 | 1.97E-08 | 0.00265859 | 159.820464 |
| rs11768365 | A | G | 0.1968 | 0.0152 | -0.0837 | 3.88E-08 | 0.00221478 | 133.081609 |
| rs78771661 | C | T | 0.0249 | 0.0669 | -0.3848 | 8.95E-09 | 0.00719033 | 434.218211 |
| rs4380956 | G | A | 0.6302 | 0.0127 | 0.0907 | 1.12E-12 | 0.00383433 | 230.772319 |
| rs938650 | G | A | 0.1044 | 0.0189 | -0.1074 | 1.41E-08 | 0.00215702 | 129.603384 |
| rs1887428 | G | C | 0.6282 | 0.0131 | -0.1643 | 2.46E-36 | 0.01260992 | 765.683149 |
| rs10114470 | T | C | 0.675 | 0.0137 | 0.1475 | 4.10E-27 | 0.00954556 | 577.819337 |
| rs3829110 | A | G | 0.3936 | 0.0125 | 0.1574 | 3.52E-36 | 0.01182643 | 717.539656 |
| rs1250573 | G | A | 0.2863 | 0.0138 | -0.098 | 1.11E-12 | 0.00392482 | 236.23948 |
| rs10826797 | G | T | 0.3101 | 0.0136 | -0.099 | 3.99E-13 | 0.00419361 | 252.486868 |
| rs6584282 | A | G | 0.507 | 0.0124 | -0.152 | 1.19E-34 | 0.01154974 | 700.555643 |
| rs11195128 | C | T | 0.3201 | 0.0133 | 0.0792 | 2.74E-09 | 0.0027303 | 164.143564 |
| rs2384352 | A | G | 0.3419 | 0.0131 | 0.0951 | 3.12E-13 | 0.00406988 | 245.00704 |
| rs10761659 | A | G | 0.503 | 0.0126 | 0.1585 | 2.30E-36 | 0.01256067 | 762.654593 |
| rs7918084 | C | T | 0.5398 | 0.0125 | 0.071 | 1.38E-08 | 0.00250453 | 150.536101 |
| rs111456533 | G | A | 0.167 | 0.017 | -0.1031 | 1.18E-09 | 0.00295739 | 177.83633 |
| rs11221335 | T | C | 0.2445 | 0.0148 | 0.0827 | 2.44E-08 | 0.0025267 | 151.872267 |
| rs11236797 | C | A | 0.4503 | 0.0125 | 0.1488 | 7.19E-33 | 0.01096134 | 664.470463 |
| rs11066188 | G | A | 0.4135 | 0.013 | 0.0874 | 1.76E-11 | 0.00370507 | 222.963554 |
| rs117981694 | G | A | 0.0229 | 0.0411 | 0.3452 | 4.53E-17 | 0.00533269 | 321.435342 |
| rs12825700 | G | A | 0.3598 | 0.0127 | 0.1324 | 1.27E-25 | 0.00807575 | 488.123474 |
| rs3897234 | T | C | 0.2336 | 0.0145 | 0.0971 | 1.90E-11 | 0.00337596 | 203.091074 |
| rs140933577 | T | C | 0.0368 | 0.0305 | -0.1857 | 1.13E-09 | 0.00244466 | 146.928655 |
| rs194746 | C | T | 0.4712 | 0.0124 | 0.0833 | 1.84E-11 | 0.00345793 | 208.039834 |
| rs3850378 | T | C | 0.1054 | 0.0207 | 0.1536 | 1.10E-13 | 0.0044492 | 267.943924 |
| rs1864239 | A | G | 0.0189 | 0.1782 | 1.3366 | 6.27E-14 | 0.06625337 | 4254.06724 |
| rs56062135 | C | T | 0.2147 | 0.0145 | 0.1382 | 1.37E-21 | 0.00644041 | 388.637955 |
| rs7190426 | A | C | 0.1879 | 0.0155 | -0.0872 | 2.06E-08 | 0.00232059 | 139.454861 |
| rs28374519 | G | A | 0.4612 | 0.0137 | -0.1105 | 6.55E-16 | 0.00606836 | 366.049931 |
| rs9934775 | C | T | 0.1581 | 0.0172 | -0.1116 | 8.77E-11 | 0.00331551 | 199.442851 |
| rs8056255 | T | A | 0.0368 | 0.0327 | 0.2765 | 2.99E-17 | 0.00541982 | 326.715821 |
| rs11548656 | A | G | 0.0398 | 0.0362 | -0.2374 | 5.18E-11 | 0.00430761 | 259.379959 |
| rs749910 | G | A | 0.2256 | 0.0138 | 0.1961 | 7.83E-46 | 0.01343661 | 816.563632 |
| rs2301127 | G | A | 0.5209 | 0.0126 | 0.0783 | 4.96E-10 | 0.00306009 | 184.030783 |
| rs16940202 | T | C | 0.1889 | 0.0169 | 0.113 | 2.50E-11 | 0.00391285 | 235.516486 |
| rs12936409 | C | T | 0.4722 | 0.0124 | 0.1406 | 7.73E-30 | 0.00985362 | 596.65325 |
| rs744166 | A | G | 0.4145 | 0.0126 | -0.1109 | 1.34E-18 | 0.00596959 | 360.056171 |
| rs714910 | A | C | 0.3201 | 0.0139 | -0.0959 | 6.23E-12 | 0.00400311 | 240.971317 |
| rs113846785 | C | CG | 0.1352 | 0.018 | -0.1319 | 2.47E-13 | 0.00406829 | 244.910727 |
| rs1319951 | C | G | 0.2475 | 0.0147 | -0.0851 | 7.50E-09 | 0.00269756 | 162.169564 |
| rs80262450 | G | A | 0.0895 | 0.019 | 0.1581 | 1.04E-16 | 0.00407377 | 245.242062 |
| rs4807569 | A | C | 0.1988 | 0.0152 | 0.1281 | 4.24E-17 | 0.0052274 | 315.055574 |
| rs7256518 | G | A | 0.9503 | 0.0276 | -0.1665 | 1.63E-09 | 0.00261864 | 157.412693 |
| rs62126610 | A | G | 0.174 | 0.0166 | 0.1407 | 2.60E-17 | 0.00569046 | 343.124151 |
| rs11669299 | C | T | 0.172 | 0.0157 | -0.1107 | 1.84E-12 | 0.00349047 | 210.004196 |
| rs6062496 | G | A | 0.5994 | 0.0129 | 0.137 | 2.83E-26 | 0.00901361 | 545.32641 |
| rs4256018 | T | G | 0.2783 | 0.0138 | 0.0786 | 1.23E-08 | 0.00248168 | 149.159021 |
| rs6017342 | A | C | 0.5199 | 0.0135 | 0.1156 | 1.07E-17 | 0.0066711 | 402.651686 |
| rs6063502 | A | G | 0.4016 | 0.0134 | -0.0734 | 4.55E-08 | 0.00258945 | 155.653487 |
| rs154873 | G | A | 0.3519 | 0.0132 | -0.0813 | 7.38E-10 | 0.0030149 | 181.304721 |
| rs1297264 | A | G | 0.4384 | 0.0126 | -0.1462 | 3.98E-31 | 0.01052501 | 637.738991 |
| rs2836881 | G | T | 0.2575 | 0.0146 | -0.1643 | 1.96E-29 | 0.01032236 | 625.331723 |
| rs2838517 | T | C | 0.6163 | 0.0125 | -0.128 | 1.83E-24 | 0.00774879 | 468.206752 |
| rs2413583 | C | T | 0.165 | 0.0171 | -0.1732 | 4.60E-24 | 0.00826602 | 499.719621 |
| rs5754100 | T | C | 0.1839 | 0.016 | 0.1293 | 7.14E-16 | 0.00501825 | 302.386532 |
| rs5763793 | G | T | 0.3628 | 0.013 | 0.0734 | 1.47E-08 | 0.00249095 | 149.717899 |
