# Supplementary material for: Evidence for genetic correlation between appendix and inflammatory bowel disease: A bidirectional Mendelian randomization study
Source: PLoS One. 2026 Feb 11;21(2):e0342541. doi: 10.1371/journal.pone.0342541 (PMC12893558; doi:10.1371/journal.pone.0342541)
Supplement: S7 Table — (DOCX) [file pone.0342541.s015.docx]

**Table S7: genetic variants used as instrumental variables for CD.**

| SNP | other allele | effect allele | | eaf | se | beta | pval | R2 | F |
| --- | --- | --- | --- | --- | --- | --- | --- | --- | --- |
| rs12131079 | C | T | 0.6779 | | 0.0174 | -0.1088 | 3.99E-10 | 0.00516945 | 209.224216 |
| rs35730213 | G | C | 0.2744 | | 0.0181 | -0.1166 | 1.17E-10 | 0.00541388 | 219.170955 |
| rs3122605 | G | A | 0.8489 | | 0.0227 | -0.1748 | 1.24E-14 | 0.00783852 | 318.103468 |
| rs114802258 | C | T | 0.0646 | | 0.0384 | -0.2245 | 5.11E-09 | 0.00609106 | 246.753254 |
| rs4316387 | T | C | 0.8042 | | 0.0189 | -0.1292 | 7.74E-12 | 0.00525693 | 212.783414 |
| rs6679677 | C | A | 0.0915 | | 0.0286 | -0.2275 | 1.77E-15 | 0.00860476 | 349.469205 |
| rs6704109 | C | T | 0.2336 | | 0.0181 | 0.1748 | 5.10E-22 | 0.0109406 | 445.385147 |
| rs7517847 | T | G | 0.4205 | | 0.0165 | -0.3447 | 5.84E-97 | 0.05790713 | 2474.88601 |
| rs11378157 | A | AG | 0.7793 | | 0.0185 | -0.1376 | 9.29E-14 | 0.00651289 | 263.954186 |
| rs11683692 | T | C | 0.0547 | | 0.038 | -0.2144 | 1.75E-08 | 0.00475375 | 192.319318 |
| rs4343432 | A | G | 0.4473 | | 0.0162 | 0.1123 | 3.50E-12 | 0.0062356 | 252.645374 |
| rs11677002 | T | C | 0.4592 | | 0.0163 | -0.1124 | 4.57E-12 | 0.00627482 | 254.244638 |
| rs34004493 | A | G | 0.2465 | | 0.0179 | 0.1258 | 2.00E-12 | 0.00587884 | 238.105297 |
| rs3816234 | G | A | 0.5427 | | 0.0162 | 0.2704 | 1.51E-62 | 0.03629146 | 1516.26671 |
| rs55946629 | C | A | 0.1233 | | 0.0231 | 0.1755 | 2.85E-14 | 0.00665884 | 269.908651 |
| rs7608697 | A | C | 0.3628 | | 0.0163 | 0.1229 | 4.03E-14 | 0.00698356 | 283.163509 |
| rs6740847 | A | G | 0.5447 | | 0.0161 | -0.104 | 9.72E-11 | 0.00536478 | 217.172476 |
| rs1583792 | C | T | 0.5507 | | 0.016 | -0.0882 | 3.26E-08 | 0.00384963 | 155.600388 |
| rs56116661 | C | T | 0.1938 | | 0.0212 | -0.1312 | 5.67E-10 | 0.00537891 | 217.747655 |
| rs6808936 | A | G | 0.4235 | | 0.0161 | 0.0904 | 1.93E-08 | 0.00399043 | 161.314345 |
| rs9836291 | G | A | 0.3241 | | 0.017 | 0.1722 | 3.77E-24 | 0.01299145 | 529.973033 |
| rs2581828 | C | G | 0.6103 | | 0.0162 | -0.0941 | 6.46E-09 | 0.00421195 | 170.307206 |
| rs73243877 | A | G | 0.1799 | | 0.0212 | 0.1164 | 4.12E-08 | 0.00399792 | 161.618326 |
| rs13107325 | C | T | 0.0795 | | 0.0284 | 0.2006 | 1.66E-12 | 0.00588956 | 238.54211 |
| rs62324212 | C | A | 0.4642 | | 0.0163 | 0.106 | 8.02E-11 | 0.0055892 | 226.308391 |
| rs6579807 | C | T | 0.16 | | 0.0244 | 0.1993 | 3.44E-16 | 0.01067687 | 434.532851 |
| rs755374 | C | T | 0.327 | | 0.0174 | 0.1969 | 1.38E-29 | 0.01706413 | 698.998075 |
| rs6451494 | T | C | 0.5895 | | 0.0166 | 0.2605 | 8.26E-56 | 0.03284297 | 1367.29538 |
| rs112856973 | T | C | 0.1312 | | 0.0243 | -0.1612 | 3.61E-11 | 0.00592398 | 239.944632 |
| rs6873866 | T | C | 0.5954 | | 0.0164 | -0.1314 | 1.35E-15 | 0.0083187 | 337.753788 |
| rs2188962 | C | T | 0.3867 | | 0.016 | 0.2004 | 5.59E-36 | 0.01904902 | 781.883732 |
| rs181826 | C | A | 0.6203 | | 0.0167 | 0.1162 | 3.24E-12 | 0.0063604 | 257.73456 |
| rs1012636 | G | T | 0.7942 | | 0.0198 | 0.1291 | 7.01E-11 | 0.00544826 | 220.570419 |
| rs1321859 | C | T | 0.3628 | | 0.0172 | -0.1049 | 1.18E-09 | 0.00508773 | 205.899906 |
| rs73516754 | A | C | 0.3151 | | 0.0169 | 0.1423 | 4.04E-17 | 0.00874008 | 355.013392 |
| rs35171809 | A | G | 0.4473 | | 0.0159 | 0.1566 | 9.07E-23 | 0.01212556 | 494.216275 |
| rs111281598 | T | C | 0.0586 | | 0.0316 | 0.2745 | 4.17E-18 | 0.00831355 | 337.54295 |
| rs6941902 | T | C | 0.1133 | | 0.0273 | 0.163 | 2.39E-09 | 0.00533841 | 216.099316 |
| rs7753014 | C | G | 0.4801 | | 0.0163 | -0.0989 | 1.39E-09 | 0.00488286 | 197.568095 |
| rs145568234 | T | G | 0.0139 | | 0.0633 | 0.8602 | 4.31E-42 | 0.02028452 | 833.645819 |
| rs9482770 | T | C | 0.4105 | | 0.0162 | 0.0987 | 1.01E-09 | 0.00471478 | 190.735105 |
| rs9501641 | C | T | 0.0417 | | 0.0432 | 0.3027 | 2.57E-12 | 0.00732306 | 297.030717 |
| rs9258357 | T | C | 0.829 | | 0.0216 | -0.1179 | 5.00E-08 | 0.00394102 | 159.308925 |
| rs212409 | G | A | 0.5586 | | 0.0162 | -0.1096 | 1.49E-11 | 0.00592358 | 239.928318 |
| rs9656588 | T | C | 0.6849 | | 0.0173 | 0.1183 | 8.73E-12 | 0.00604053 | 244.693986 |
| rs938650 | G | A | 0.1044 | | 0.0247 | -0.1747 | 1.65E-12 | 0.0057073 | 231.117619 |
| rs4380956 | G | A | 0.6302 | | 0.0165 | 0.132 | 1.15E-15 | 0.00812126 | 329.671571 |
| rs79832570 | T | C | 0.0934 | | 0.0344 | 0.2234 | 8.90E-11 | 0.00845199 | 343.211708 |
| rs10114470 | T | C | 0.675 | | 0.0177 | 0.1687 | 1.76E-21 | 0.01248669 | 509.121285 |
| rs1887428 | G | C | 0.6282 | | 0.0169 | -0.166 | 8.54E-23 | 0.01287222 | 525.045612 |
| rs4077515 | C | T | 0.3966 | | 0.0162 | 0.1848 | 3.14E-30 | 0.01634526 | 669.061709 |
| rs10884966 | G | A | 0.3201 | | 0.0171 | 0.1131 | 4.13E-11 | 0.00556783 | 225.438285 |
| rs61839660 | C | T | 0.0696 | | 0.0261 | 0.1468 | 1.98E-08 | 0.00279101 | 112.691661 |
| rs2002695 | A | G | 0.2525 | | 0.0189 | -0.1293 | 8.31E-12 | 0.00631102 | 255.720809 |
| rs10822050 | T | C | 0.3767 | | 0.0162 | 0.1827 | 2.35E-29 | 0.01567472 | 641.177268 |
| rs2675670 | G | C | 0.5865 | | 0.0161 | 0.1074 | 2.89E-11 | 0.00559477 | 226.535154 |
| rs1148246 | C | T | 0.6392 | | 0.0167 | -0.1323 | 2.09E-15 | 0.00807334 | 327.710481 |
| rs1250573 | G | A | 0.2863 | | 0.0179 | -0.1522 | 1.92E-17 | 0.00946665 | 384.808061 |
| rs6584282 | A | G | 0.507 | | 0.016 | -0.1658 | 3.44E-25 | 0.01374213 | 561.022605 |
| rs11236797 | C | A | 0.4503 | | 0.0161 | 0.176 | 8.51E-28 | 0.01533497 | 627.063352 |
| rs28999107 | G | T | 0.4374 | | 0.0178 | 0.1083 | 1.06E-09 | 0.00577252 | 233.774195 |
| rs77566919 | G | A | 0.6879 | | 0.0185 | -0.1089 | 4.13E-09 | 0.00509219 | 206.081455 |
| rs34635748 | C | T | 0.0229 | | 0.0504 | 0.4794 | 1.95E-21 | 0.01028491 | 418.41503 |
| rs1373904 | A | G | 0.2256 | | 0.0189 | 0.141 | 9.11E-14 | 0.00694661 | 281.654684 |
| rs194746 | C | T | 0.4712 | | 0.0161 | 0.0975 | 1.24E-09 | 0.00473736 | 191.6528 |
| rs3850378 | T | C | 0.1054 | | 0.0267 | 0.199 | 8.31E-14 | 0.00746802 | 302.954957 |
| rs72743461 | C | A | 0.2147 | | 0.0187 | 0.1684 | 2.26E-19 | 0.00956273 | 388.75121 |
| rs2021511 | C | T | 0.2962 | | 0.0182 | -0.1082 | 2.63E-09 | 0.00488111 | 197.497127 |
| rs42861 | A | G | 0.3946 | | 0.0167 | 0.1243 | 8.87E-14 | 0.00738196 | 299.437718 |
| rs2076756 | A | G | 0.2256 | | 0.0174 | 0.385 | 1.80E-108 | 0.05179119 | 2199.22076 |
| rs7195228 | C | G | 0.1879 | | 0.0209 | -0.1327 | 2.09E-10 | 0.00537413 | 217.553111 |
| rs72798422 | T | C | 0.0378 | | 0.0382 | 0.5495 | 6.05E-47 | 0.02196456 | 904.242401 |
| rs10492862 | C | A | 0.2823 | | 0.0176 | 0.1067 | 1.26E-09 | 0.00461331 | 186.611218 |
| rs2948542 | A | G | 0.6173 | | 0.0163 | 0.1016 | 5.15E-10 | 0.00487722 | 197.338762 |
| rs714910 | A | C | 0.3201 | | 0.0181 | -0.1531 | 2.49E-17 | 0.01020261 | 415.032115 |
| rs12936409 | C | T | 0.4722 | | 0.016 | 0.1426 | 4.31E-19 | 0.01013595 | 412.292828 |
| rs744166 | A | G | 0.4145 | | 0.0162 | -0.1142 | 1.80E-12 | 0.00633015 | 256.500632 |
| rs80262450 | G | A | 0.0895 | | 0.0244 | 0.2268 | 1.34E-20 | 0.00838338 | 340.402077 |
| rs144309607 | C | T | 0.0308 | | 0.047 | -0.3712 | 2.69E-15 | 0.0082264 | 333.975361 |
| rs62126620 | G | A | 0.2018 | | 0.0201 | 0.144 | 8.61E-13 | 0.00668018 | 270.779436 |
| rs4807570 | G | A | 0.1998 | | 0.0193 | 0.1811 | 6.03E-21 | 0.01048723 | 426.73321 |
| rs492602 | A | G | 0.4414 | | 0.0162 | 0.1084 | 2.33E-11 | 0.00579458 | 234.672722 |
| rs6062496 | G | A | 0.5994 | | 0.0167 | 0.1223 | 2.62E-13 | 0.00718308 | 291.311977 |
| rs3761158 | G | A | 0.4155 | | 0.0165 | -0.1098 | 2.65E-11 | 0.00585585 | 237.168921 |
| rs1297264 | A | G | 0.4384 | | 0.0163 | -0.1769 | 1.59E-27 | 0.01540931 | 630.150812 |
| rs2284553 | A | G | 0.6193 | | 0.0165 | 0.1277 | 1.14E-14 | 0.00768946 | 312.007519 |
| rs2838517 | T | C | 0.6163 | | 0.0162 | -0.1456 | 2.03E-19 | 0.01002621 | 407.783766 |
| rs2143178 | T | C | 0.167 | | 0.0223 | -0.2087 | 6.84E-21 | 0.01211815 | 493.910522 |
| rs5754100 | T | C | 0.1839 | | 0.0206 | 0.1687 | 3.02E-16 | 0.00854251 | 346.919 |
