# Supplementary material for: Evidence for genetic correlation between appendix and inflammatory bowel disease: A bidirectional Mendelian randomization study
Source: PLoS One. 2026 Feb 11;21(2):e0342541. doi: 10.1371/journal.pone.0342541 (PMC12893558; doi:10.1371/journal.pone.0342541)
Supplement: S8 Table — (DOCX) [file pone.0342541.s016.docx]

**Table S8: genetic variants used as instrumental variables for UC.**

| SNP | other allele | effect allele | eaf | se | beta | pval | R2 | F |
| --- | --- | --- | --- | --- | --- | --- | --- | --- |
| rs10272963 | T | C | 0.455467 | 0.016 | -0.1512 | 4.11E-21 | 0.01134004 | 527.315575 |
| rs10408351 | A | G | 0.183473 | 0.0204 | 0.1548 | 2.92E-14 | 0.00717984 | 332.465724 |
| rs10761659 | G | A | 0.52802 | 0.016 | 0.1276 | 1.33E-15 | 0.00811531 | 376.137794 |
| rs10817678 | A | G | 0.678839 | 0.017 | 0.1332 | 4.42E-15 | 0.00773621 | 358.429488 |
| rs11209026 | A | G | 0.0456299 | 0.0358 | -0.483 | 1.99E-41 | 0.02031845 | 953.473254 |
| rs1131095 | C | T | 0.411965 | 0.0168 | 0.1593 | 2.18E-21 | 0.0122949 | 572.269491 |
| rs113846785 | CG | C | 0.1431 | 0.0229 | -0.1627 | 1.15E-12 | 0.00649195 | 300.404429 |
| rs113986290 | T | C | 0.0182714 | 0.0531 | -0.3066 | 7.59E-09 | 0.00337239 | 155.563424 |
| rs11645239 | G | C | 0.160382 | 0.02 | -0.1174 | 4.14E-09 | 0.00371196 | 171.285849 |
| rs11651246 | G | T | 0.185268 | 0.0219 | 0.147 | 2.01E-11 | 0.00652349 | 301.873572 |
| rs12825700 | A | G | 0.32278 | 0.0161 | 0.1889 | 7.33E-32 | 0.0156002 | 728.553811 |
| rs12936409 | T | C | 0.514831 | 0.0158 | 0.1365 | 5.62E-18 | 0.00930793 | 431.933799 |
| rs1317209 | A | G | 0.220176 | 0.0203 | 0.1818 | 2.90E-19 | 0.0113497 | 527.76972 |
| rs13200059 | A | G | 0.0488116 | 0.0436 | 0.2944 | 1.48E-11 | 0.00804813 | 372.998806 |
| rs1359946 | A | G | 0.176467 | 0.0202 | 0.1571 | 6.58E-15 | 0.00717343 | 332.166881 |
| rs137845 | G | A | 0.569785 | 0.0158 | 0.1011 | 1.50E-10 | 0.00501105 | 231.533297 |
| rs16940186 | C | T | 0.145411 | 0.0214 | 0.1357 | 2.18E-10 | 0.00457661 | 211.368009 |
| rs1736161 | A | G | 0.398844 | 0.0161 | -0.1227 | 2.22E-14 | 0.00721954 | 334.31741 |
| rs17656349 | T | C | 0.607584 | 0.0159 | 0.09 | 1.54E-08 | 0.0038625 | 178.259056 |
| rs17715902 | A | G | 0.351665 | 0.0166 | 0.0974 | 4.62E-09 | 0.0043259 | 199.738672 |
| rs1811711 | G | C | 0.148141 | 0.0223 | -0.1299 | 6.09E-09 | 0.00425884 | 196.629046 |
| rs1887428 | C | G | 0.616203 | 0.0166 | -0.167 | 9.65E-24 | 0.01319132 | 614.551392 |
| rs2045241 | A | G | 0.392643 | 0.0169 | -0.1063 | 2.83E-10 | 0.00538938 | 249.108287 |
| rs2212434 | T | C | 0.405242 | 0.0159 | 0.1252 | 2.80E-15 | 0.00755603 | 350.017889 |
| rs2816954 | A | T | 0.81276 | 0.0229 | 0.1375 | 1.80E-09 | 0.00575435 | 266.075871 |
| rs2836881 | T | G | 0.247334 | 0.0186 | -0.2217 | 1.11E-32 | 0.01829985 | 856.981582 |
| rs28383224 | G | A | 0.625696 | 0.0165 | -0.1468 | 4.65E-19 | 0.01009415 | 468.790616 |
| rs2838517 | C | T | 0.3186 | 0.016 | -0.1177 | 1.78E-13 | 0.00601493 | 278.197874 |
| rs3024493 | A | C | 0.156104 | 0.0209 | 0.21 | 7.46E-24 | 0.01161907 | 540.443172 |
| rs3734851 | A | G | 0.0141207 | 0.0584 | 0.5033 | 6.58E-18 | 0.00705284 | 326.543119 |
| rs3812565 | C | T | 0.37036 | 0.016 | 0.1335 | 6.50E-17 | 0.00831206 | 385.333465 |
| rs3820330 | A | C | 0.293937 | 0.0178 | -0.1587 | 3.91E-19 | 0.01045398 | 485.677973 |
| rs4654925 | C | G | 0.503578 | 0.0159 | -0.2217 | 2.61E-44 | 0.02457419 | 1158.21117 |
| rs4676408 | A | G | 0.631831 | 0.0167 | 0.1433 | 1.19E-17 | 0.00955368 | 443.447719 |
| rs4728142 | A | G | 0.420958 | 0.0158 | 0.0995 | 3.23E-10 | 0.00482642 | 222.961048 |
| rs4993442 | T | G | 0.711456 | 0.0179 | -0.0988 | 3.54E-08 | 0.00400778 | 184.991119 |
| rs55905347 | A | G | 0.424761 | 0.0166 | 0.1054 | 2.09E-10 | 0.0054288 | 250.940721 |
| rs56062135 | T | C | 0.262157 | 0.0184 | 0.1078 | 4.66E-09 | 0.00449565 | 207.612081 |
| rs6017342 | C | A | 0.559163 | 0.017 | 0.1944 | 3.95E-30 | 0.01863112 | 872.789564 |
| rs6062496 | A | G | 0.64519 | 0.0163 | 0.1359 | 8.97E-17 | 0.00845575 | 392.051514 |
| rs62180181 | T | C | 0.378764 | 0.0171 | 0.1226 | 8.08E-13 | 0.00707353 | 327.508067 |
| rs6658353 | C | G | 0.494944 | 0.016 | -0.1569 | 1.17E-22 | 0.01230755 | 572.865398 |
| rs67111717 | G | A | 0.360294 | 0.0171 | 0.0944 | 3.27E-08 | 0.00410782 | 189.62778 |
| rs6889364 | A | G | 0.193595 | 0.0228 | 0.1318 | 7.87E-09 | 0.00542386 | 250.710759 |
| rs6933404 | C | T | 0.18927 | 0.0188 | 0.1486 | 2.69E-15 | 0.00677682 | 313.676249 |
| rs7203363 | A | T | 0.820284 | 0.0189 | 0.1071 | 1.41E-08 | 0.00338189 | 156.003375 |
| rs72704802 | T | C | 0.12633 | 0.0206 | -0.1223 | 2.89E-09 | 0.00330169 | 152.291602 |
| rs7523335 | A | G | 0.265783 | 0.021 | -0.1389 | 3.42E-11 | 0.00752985 | 348.796013 |
| rs7544646 | G | C | 0.475468 | 0.016 | -0.1168 | 2.53E-13 | 0.0068047 | 314.975774 |
| rs755374 | T | C | 0.299928 | 0.0171 | 0.1714 | 9.73E-24 | 0.01233705 | 574.255857 |
| rs7554511 | A | C | 0.212908 | 0.0178 | -0.1448 | 4.27E-16 | 0.00702724 | 325.349472 |
| rs7608697 | C | A | 0.370039 | 0.0161 | 0.1597 | 3.03E-23 | 0.01189052 | 553.221145 |
| rs78064630 | A | G | 0.059641 | 0.0308 | 0.1759 | 1.08E-08 | 0.00347057 | 160.107985 |
| rs79051659 | A | G | 0.069621 | 0.0264 | 0.1605 | 1.30E-09 | 0.00333718 | 153.934092 |
| rs7911117 | G | T | 0.136773 | 0.0239 | -0.1342 | 1.84E-08 | 0.00425266 | 196.342404 |
| rs7911680 | C | A | 0.529894 | 0.0159 | -0.1525 | 6.71E-22 | 0.01158656 | 538.913027 |
| rs798506 | C | T | 0.337922 | 0.0179 | -0.1206 | 1.47E-11 | 0.00650804 | 301.154061 |
| rs9260809 | G | A | 0.404441 | 0.0184 | -0.1102 | 1.96E-09 | 0.00585023 | 270.53545 |
| rs9267798 | C | G | 0.068899 | 0.028 | 0.2486 | 6.54E-19 | 0.00792943 | 367.45337 |
| rs9271176 | G | A | 0.579337 | 0.0173 | -0.3495 | 4.20E-91 | 0.05953741 | 2910.39045 |
| rs9611131 | C | T | 0.118251 | 0.0227 | -0.1494 | 5.11E-11 | 0.00465459 | 214.985918 |
| rs989960 | T | C | 0.507834 | 0.016 | -0.1214 | 3.28E-14 | 0.00736717 | 341.204666 |
