# Supplementary material for: Evidence for genetic correlation between appendix and inflammatory bowel disease: A bidirectional Mendelian randomization study
Source: PLoS One. 2026 Feb 11;21(2):e0342541. doi: 10.1371/journal.pone.0342541 (PMC12893558; doi:10.1371/journal.pone.0342541)
Supplement: S9 Table — (DOCX) [file pone.0342541.s017.docx]

**Table S10: Multivariable MR estimates for UC and CD using IVW method.**

| exposure | outcome | nsnp | se | pval | OR | Lower CI | Upper CI |
| --- | --- | --- | --- | --- | --- | --- | --- |
| Crohn's disease | Appendectomy | 69 | 0.010 | 0.889 | 0.999 | 0.978 | 1.019 |
| Ulcerative colitis | Appendectomy | 45 | 0.013 | 0.002* | 0.961 | 0.937 | 0.986 |
| Crohn's disease | Acute appendicitis | 69 | 0.013 | 0.884 | 1.002 | 0.976 | 1.029 |
| Ulcerative colitis | Acute appendicitis | 45 | 0.017 | 1.083E-04* | 0.937 | 0.907 | 0.969 |

IVW: Inverse variance weighted; nsnp= number of single nucleotide polymorphisms, beta: beta-coefficient (in standard deviation units), se: standard error, se: standard error.
