# Supplementary material for: Evidence for genetic correlation between appendix and inflammatory bowel disease: A bidirectional Mendelian randomization study
Source: PLoS One. 2026 Feb 11;21(2):e0342541. doi: 10.1371/journal.pone.0342541 (PMC12893558; doi:10.1371/journal.pone.0342541)
Supplement: S11 Table — (DOCX) [file pone.0342541.s019.docx]

**Table S11: Heterogeneity and pleiotropy analyses of IBD and it’s subtypes with appendicitis and appendectomy.**

| Exposure | Outcome | MR Egger | | | | IVW | |
| --- | --- | --- | --- | --- | --- | --- | --- |
|  |  | Intercept | Pleiotropy  p-value | Cochran’s Q  statistic | Heterogeneity  p-value | Cochran’s Q  statistic | Heterogeneity  p-value |
| IBD | Acute appendicitis | -0.003 | 0.183 | 178.600 | 2.366e-07 | 182.054 | 1.40e-07 |
|  | Appendectomy | 0.001 | 0.750 | 139.947 | 0.001 | 140.098 | 0.002 |
| CD | Appendicitis | -0.009 | 0.043 | 147.7301 | 2.545e-07 | 156.533 | 3.312e-08 |
|  | Appendectomy | -0.006 | 0.100 | 85.652 | 0.167 | 88.860 | 0.131 |
| UC | Appendicitis | 0.006 | 0.259 | 96.168 | 9.496e-05 | 98.671 | 7.080e-05 |
|  | Appendectomy | 0.009 | 0.100 | 56.813 | 0.207 | 60.070 | 0.156 |

IVW: Inverse variance weighted; IBD: Inflammatory Bowel Disease; CD: Crohn’s Disease; UC: Ulcerative Colitis.
