# Supplementary material for: Evidence for genetic correlation between appendix and inflammatory bowel disease: A bidirectional Mendelian randomization study
Source: PLoS One. 2026 Feb 11;21(2):e0342541. doi: 10.1371/journal.pone.0342541 (PMC12893558; doi:10.1371/journal.pone.0342541)
Supplement: S12 Table — (DOCX) [file pone.0342541.s020.docx]

**Table S12. LDSC Regression Estimates of IBD, UC, CD, and acute appendicitis.**

| Trait | mean_chisq | lambda_gc | intercept | intercept_se | ratio | ratio_se | h2_observed | h2_observed_se | h2_Z | h2_p |
| --- | --- | --- | --- | --- | --- | --- | --- | --- | --- | --- |
| Acute appendicitis | 1.166534895 | 1.142851278 | 1.08871423 | 0.011942205 | 0.53270656 | 0.07170993 | 0.010353145 | 0.001879858 | 5.50740779 | 3.64E-08 |
| IBD | 1.471732501 | 1.296497642 | 1.15697075 | 0.014689344 | 0.33275373 | 0.03113914 | 0.258761704 | 0.025747405 | 10.0500109 | 9.19E-24 |
| CD | 1.413160249 | 1.262446476 | 1.13065246 | 0.013632123 | 0.31622707 | 0.03299476 | 0.339025847 | 0.034798122 | 9.74264781 | 1.98E-22 |
| UC | 1.314055181 | 1.212451883 | 1.11433632 | 0.015614965 | 0.36406443 | 0.04972045 | 0.215536707 | 0.027026715 | 7.97495015 | 1.52E-15 |
