# Supplementary material for: Evidence for genetic correlation between appendix and inflammatory bowel disease: A bidirectional Mendelian randomization study
Source: PLoS One. 2026 Feb 11;21(2):e0342541. doi: 10.1371/journal.pone.0342541 (PMC12893558; doi:10.1371/journal.pone.0342541)
Supplement: S13 Table — (DOCX) [file pone.0342541.s021.docx]

**Table S13.** **Genetic Correlation Estimates between IBD and acute appendicitis from LDSC Regression.**

| Trait 1 | Trait 2 | rg | rg_se | rg_p |
| --- | --- | --- | --- | --- |
| Acute appendicitis | IBD | -0.1616012 | 0.06838992 | 0.01813067 |
| Acute appendicitis | CD | -0.0994235 | 0.06974028 | 0.15397651 |
| Acute appendicitis | UC | -0.2052475 | 0.07344349 | 0.00519589 |
